# Species and strain-specific phenotypes shape performance and output of a five-species yeast consortium

**DOI:** 10.1128/spectrum.02819-26

**Published:** 2026-09-24

**Authors:** Fanzhi Wang, Jeanne Célina Hélène Laforge, Mathabatha Evodia Setati, Florian Franz Bauer

**Affiliations:** 1 South African Grape and Wine Research Institute, Stellenbosch University 26697, Stellenbosch, South Africa; Connecticut Agricultural Experiment Station, New Haven, Connecticut, USA

**Keywords:** wine fermentation, multi-species consortium, yeast-yeast interaction, synthetic community, non-*Saccharomyces* yeasts

## Abstract

Spontaneous wine alcoholic fermentation is initiated by complex yeast communities, which shape the fermentation process and fermentation outcome. While this yeast ecosystem has been described extensively, the biotic factors impacting population dynamics, and the relative ecological importance of the taxonomic levels of species and of strains in defining these parameters remain poorly resolved. Here, we established a consortium of five yeast species that are commonly identified as major contributors to spontaneous wine fermentations: *Saccharomyces cerevisiae*, *Lachancea thermotolerans*, *Hanseniaspora uvarum*, *Starmerella bacillaris*, and *Torulaspora delbrueckii*. Each species in this consortium was represented by two phenotypically diverging strains. Two systematic consortium perturbations, single-species drop-out, and single-strain substitutions were applied, and fermentation kinetics, sugar utilization, population dynamics, primary metabolites, and major volatile compounds were characterized. Both species drop-out and strain substitution produced significant impacts on population dynamics, growth behaviors, and metabolic outputs. Two-way analysis confirmed that the non-*Saccharomyces* substitutions affected the metabolic output more broadly than the *S. cerevisiae* strain. Sugar utilization and population data jointly revealed competitive interactions among species, while chemical outputs tracked the identity of each consortium comprising different species and strains. The data support a hierarchical interpretation in which species identity establishes consortium structure, and strain identity modulates its effect size.

**IMPORTANCE:**

Microbial communities drive important processes in food production, yet we still poorly understand how these complex communities behave. Wine fermentation offers a tractable model, as it is carried out by multiple yeast species whose combined activity determines the metabolic outcome. It is commonly assumed that knowing which species are present is sufficient to predict community function, while differences among strains of the same species are often overlooked. By establishing a defined community of five wine yeast species and systematically removing species or substituting individual strains, we show that species identity sets the basic structure of the community, while the particular strain of each species tunes the size of that effect. This distinction matters for anyone seeking to design or predict the behavior of microbial communities, and it shows that strain-level differences must be accounted for when engineering predictable microbial fermentations.

## INTRODUCTION

Alcoholic fermentation of grape must is driven by a complex microbial community in which yeasts play the central role (1–3). This wine fermentation ecosystem is characterized by complex interactions between many contributing yeast species (4–6). The initial stages of spontaneous wine fermentations are shaped by a diverse population of non-*Saccharomyces* yeast species, while *Saccharomyces cerevisiae*, typically present at low initial abundance, tends to dominate the later stages as ethanol accumulates and oxygen is depleted (7). Importantly, although non-*Saccharomyces* species are progressively outcompeted and decline before the end of fermentation, they contribute substantially and in a species-specific manner to the chemical and sensory characteristics of the final wine (8, 9).

While this broad general pattern of species succession in spontaneous wine fermentations is well established, the data reveal significant variations in the persistence and in the order of species succession between individual studies (10, 11). Understanding the drivers of such variation in ecosystem development has become a core research interest in the past decade, both for its scientific contribution to microbial ecology as for its relevance to our ability to control industrially relevant microbial ecosystems. The wine fermentation ecosystem indeed offers a tractable model in which a limited number of yeast species interact within a broadly defined matrix to generate a measurable, multi-dimensional output, and in which the interactions between these species and the contribution of individual community members can be examined experimentally.

Most studies have focused on the influence of environmental factors, including temperature (12), sulfur dioxide concentrations (4), pH (13), nutrient availability (14), ethanol concentration (15), and oxygen availability (16) on the physiology and fermentation performance of individual species or in spontaneous wine fermentation ecosystems. This evidence suggests that, while all of these factors impact the growth and fermentation performance of individual species, they are insufficient to explain the specific succession of species in individual fermentations. The data suggest that wine fermentation ecology is not only a function of environmental factors but depends to a significant degree on direct ecological interactions between species (17). To elucidate the role and mechanisms of such ecological interactions, several studies have investigated biotic interactions in binary, two-species systems (18–25), and, in a limited number of studies, in more complex, synthetic communities of more than two species (26–31). Such studies have confirmed the importance of ecological interactions between species in this ecosystem. However, the complexity of such ecological interactions, and the interactions between environmental and ecological factors, limits our ability to establish causal relationships between these parameters and growth and fermentation performance of individual species. It also means that we remain unable to predict the evolution of natural grape juice communities during fermentation (4, 14, 32).

Representative synthetic consortia of relevant species provide a tool to elucidate ecosystem-relevant functions. In wine fermentation, such multi-species approaches were previously established by Bagheri et al. (4, 28), who constructed an eight-species wine yeast consortium. The data demonstrated that ecological interactions among species, and not physico-chemical conditions alone, are a primary determinant of population dynamics and significantly affect fermentation outcomes. Such synthetic consortium-based approaches offer an opportunity for systematic manipulation and for molecular interrogation of ecological processes and of individual species contribution. Moreover, whether intraspecific variation—the phenotypic differences between strains of the same species—also measurably impacts community-level behavior can be explored within such a defined multi-species context.

To address these knowledge gaps, the present study constructed a defined five-species wine yeast consortium comprising *S. cerevisiae*, *Lachancea thermotolerans*, *Hanseniaspora uvarum*, *Starmerella bacillaris* (synonym *Candida zemplinina*), and *Torulaspora delbrueckii*, each represented by two strains of diverging phenotype. These five species are commonly reported from grape must microbial diversity surveys (33–35), and all have been identified as significant contributors to fermentation processes in spontaneous fermentations. For three species, *S. cerevisiae*, *T. delbrueckii*, and *L. thermotolerans*, genome sequence analysis of wine strains has highlighted “domestication” signatures (36–39), indicating that alcoholic fermentation was an evolutionarily relevant environment for these strains. Selected *S. cerevisiae* strains are commonly inoculated to better control the process and achieve desirable outcomes, and several hundred wine strains of this species are commercially produced (40). More recently, strains of *L. thermotolerans* and of *T. delbrueckii* have also been commercialized as co-inoculants because of their ability to impart desirable characteristics to the final product (41, 42). *S. bacillaris*, while not available commercially, is commonly described as one of the most dominant species in early fermentation and has attracted widespread biotechnological interest because of its fructophilic (preference for fructose over glucose) nature (43), while *H. uvarum* is frequently the numerically most dominant species in grape must after grape pressing and has been identified as an efficient fermentation yeast at the early stages of the process (44–46). The representative nature and reduced compositional complexity of this consortium allow a systematic evaluation of ecosystem-relevant processes, preserve sufficient ecological richness for community-level interactions to emerge, and enable the assessment of biotechnologically and oenologically relevant ecosystem outputs. In order to provide baseline data for a molecular analysis of ecosystem-relevant phenotypes and processes within this representative consortium, this study applied two complementary perturbations: single-species drop-out fermentations, in which each species was removed from the consortium in turn, and single-strain substitution, in which one strain of one species was replaced by its counterpart to assess how strain identity shapes the consortium. Fermentation kinetics, sugar utilization, population dynamics, primary metabolites, and major volatiles were characterized in parallel across all consortia. We hypothesized that species identity is the primary determinant of consortium behavior, while strain identity modulates the magnitude of that, and the present work tests this hypothesis through systematic, multi-dimensional phenotypic characterization.

## RESULTS

### Establishment of the synthetic consortium: selection of species and strains

The five species forming the synthetic consortium were selected according to the following criteria: (i) globally and consistently associated with grape juice environments in published biodiversity studies, (ii) fermentative yeast that persist in grape juice during alcoholic fermentation for a significant part of the process, (iii) known to impact the chemical profile of the final product, (iv) of direct biotechnological relevance for wine fermentation, with individual strains either already commercialized for wine fermentation (co)-inoculation or having attracted commercial interest to be developed for this purpose, and (v) easily differentiated from the other species by colony morphology or ARISA. Taken together, the five yeast species also had to combine the diversity of phenotypic traits associated with natural oenological yeast communities.

**Fig 1:**
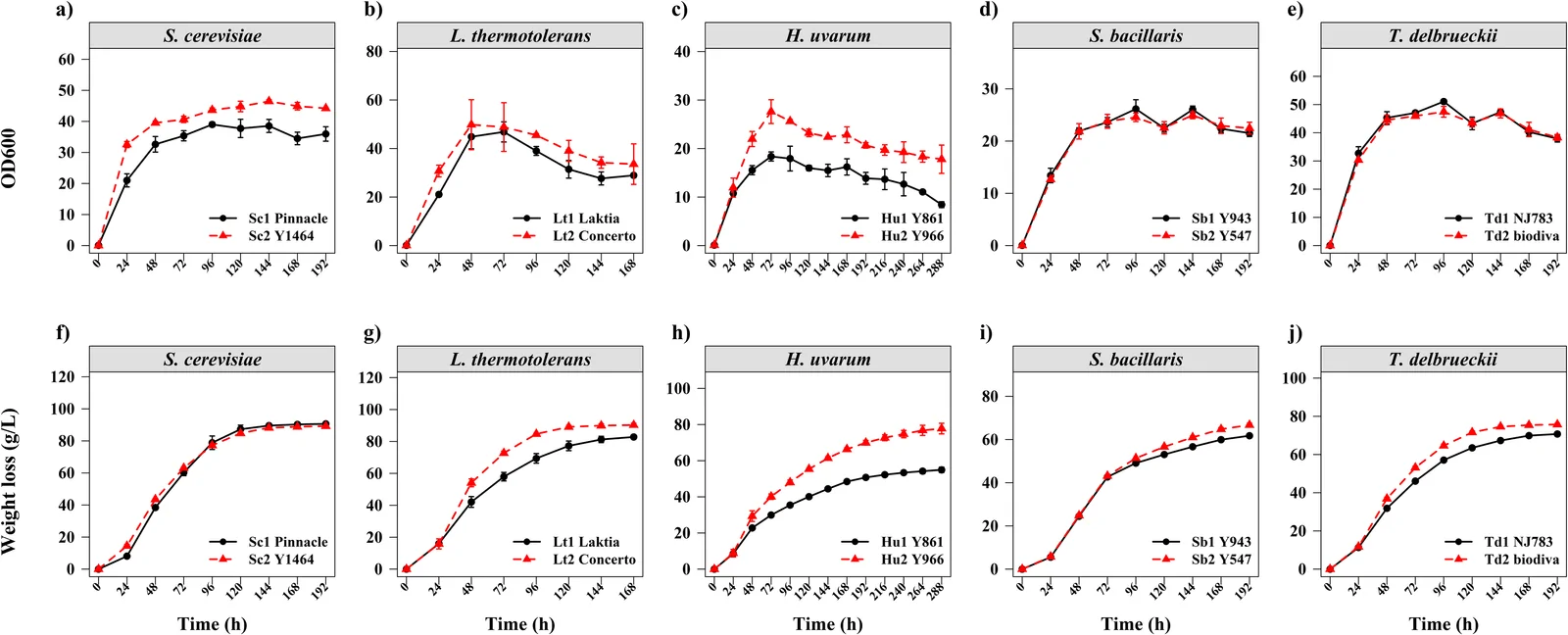
Pure-culture fermentation performance of the ten yeast strains in synthetic grape must. Each of the five species was represented by two strains, cultured individually under the same conditions. (**a–e**) Optical density (OD) growth curves for the two strains of each species. (**f–j**) Cumulative CO₂ release measured gravimetrically for the two strains of each species. Within each panel, the two strains are distinguished as follows: strain 1 is shown as a black solid line with circle symbols, and strain 2 as a red dashed line with triangle symbols. Curves represent the mean of three biological replicates; error bars indicate ±SD.

Each species in the consortium is furthermore represented by two phenotypically divergent strains, which have all been isolated from wine fermentations (Fig. 1). The primary selection criteria for phenotypic divergence between strains were growth speed and fermentation performance, both ecologically impactful traits. For three of these species, *S. cerevisiae*, *S. bacillaris*, and *L. thermotolerans*, species-specific metabolic features were added as primary strain selection criteria, with differences in hexose (glucose and fructose) preference taken into account for *S. cerevisiae* and *S. bacillaris*, and the ability to produce lactic acid for *L. thermotolerans* strains. These metabolic phenotypes are also of direct ecological and oenological relevance and produce relevant chemical impacts in the final product.

Figure 1 shows the specific phenotypes of the selected strains. For *S. cerevisiae*, the commercial strain (Sc1) has a lower initial growth rate than Sc2 and a lower early CO_2_ yield. At a later stage, this strain reaches a comparable fermentation rate to Sc2, and both strains complete fermentation. The two strains are also distinguished by their sugar consumption pattern, with Sc1 consuming glucose and fructose at a more similar rate than Sc2, which showed a stronger preference for glucose, in line with the majority of wine *S. cerevisiae* strains (47). The higher fructophilic index of Sc1 is a sought-after but unusual phenotype in commercial Sc strains. For *T. delbrueckii*, a wild isolate (Td1) and a commercial strain (Td2) were selected from the South African Grape and Wine Research Institute (SAGWRI) strain collection; the two showed comparable growth rates, but Td2 reached a significantly higher maximum CO_2_ release. The two strains of *L. thermotolerans* are both produced commercially and clearly differ in fermentation rate. Both of these strains have been characterized previously and also differ in their ability to produce lactic acid, a phenotype of significant enological interest that might also have ecological relevance (48). The strains of the two species lacking commercial representatives, *H. uvarum* and *S. bacillaris*, were wild isolates: the two *H*. *uvarum* strains were selected from a panel of seven strains as those showing the most distinct growth and fermentation profiles, and the two *S*. *bacillaris* strains from a panel of five (data not shown). The two *S*. *bacillaris* strains followed near-identical growth trajectories but significantly diverged in fermentation rate after 72 h, by which point both had consumed nearly all fructose (Fig. S1).

### Species drop-out and strain substitution affected fermentation rate but not completion

**Fig 2:**
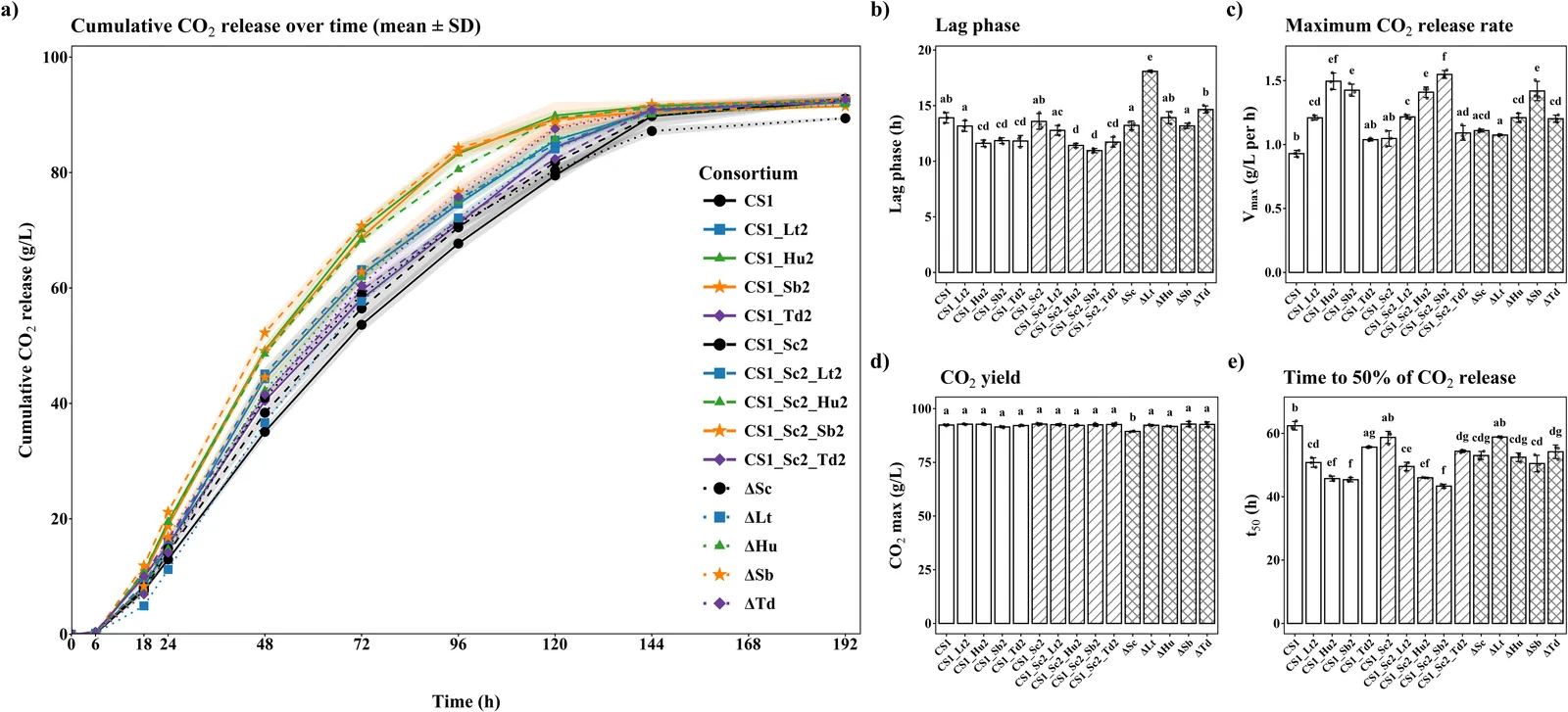
Fermentation kinetics across 15 synthetic yeast consortia in synthetic grape must. (**a**) Mean cumulative CO₂ release curves; shaded bands denote ±SEM (*n* = 3). Each color denotes the non-*Saccharomyces* species involved in each perturbation (Lt, blue; Hu, green; Sb, orange; Td, purple; CS1 baseline and all Sc-related consortia, black), while line type shows perturbation status (solid, Sc1-background; dashed, Sc2-background; dotted, drop-out). Other kinetic parameters were derived by linear interpolation between adjacent sampling points and are shown in panel **b:** Lag phase (time to 5 g L⁻¹ cumulative CO₂), (**c**) Maximum CO₂ release rate (V_max), (**d**) Maximum cumulative CO_2_ release, as well as (**e**) time required to reach 50% of the maximum cumulative CO₂ release (T50). For panels **b–e**, bars show the mean of three biological replicates with individual values overlaid; error bars indicate ±SD. Bar fill encodes perturbation status (open, Sc1-background; diagonal stripes, Sc2-background; crosshatched, drop-out). Lowercase letters indicate Tukey HSD groupings (α = 0.05).

The fermentation kinetics of all 15 consortia, representing the five species drop-out and 10 consortia with strain replacements, were monitored over 192 h by gravimetric measurement of cumulative CO_2_ release (Fig. 2). Maximum cumulative CO_2_ release converged across consortia at approximately 92.2 ± 1.0 g L⁻¹, except for fermentations conducted with the ΔSc consortium, which reached a lower final value of 89.4 ± 0.2 g L⁻¹. All fermentations reached dryness (<2 g L⁻¹ residual sugar; Fig. S2) within 192 h. In contrast, lag phase, maximum CO_2_ release rate, and t_50_ differed significantly among consortia (Fig. 2b, c and e; Tukey HSD, α = 0.05). Lag phase ranged from 11 to 14 h across most consortia, with the ΔLt consortium exhibiting the longest lag phase at 18 h. V_max ranged from 0.90 to 1.58 g L⁻¹ h⁻¹, with CS1_Hu2, CS1_Sb2, CS1_Sc2_Hu2, and CS1_Sc2_Sb2 forming the highest-rate group, and CS1 falling into the lowest. The same four consortia also formed the shortest t_50_ group, while CS1 exhibited the longest t_50_, ranging from 43.4 ± 0.6 to 62.4 ± 1.3 h across all consortia.

### Strain and species effects modulated glucose and fructose consumption across consortia

**Fig 3:**
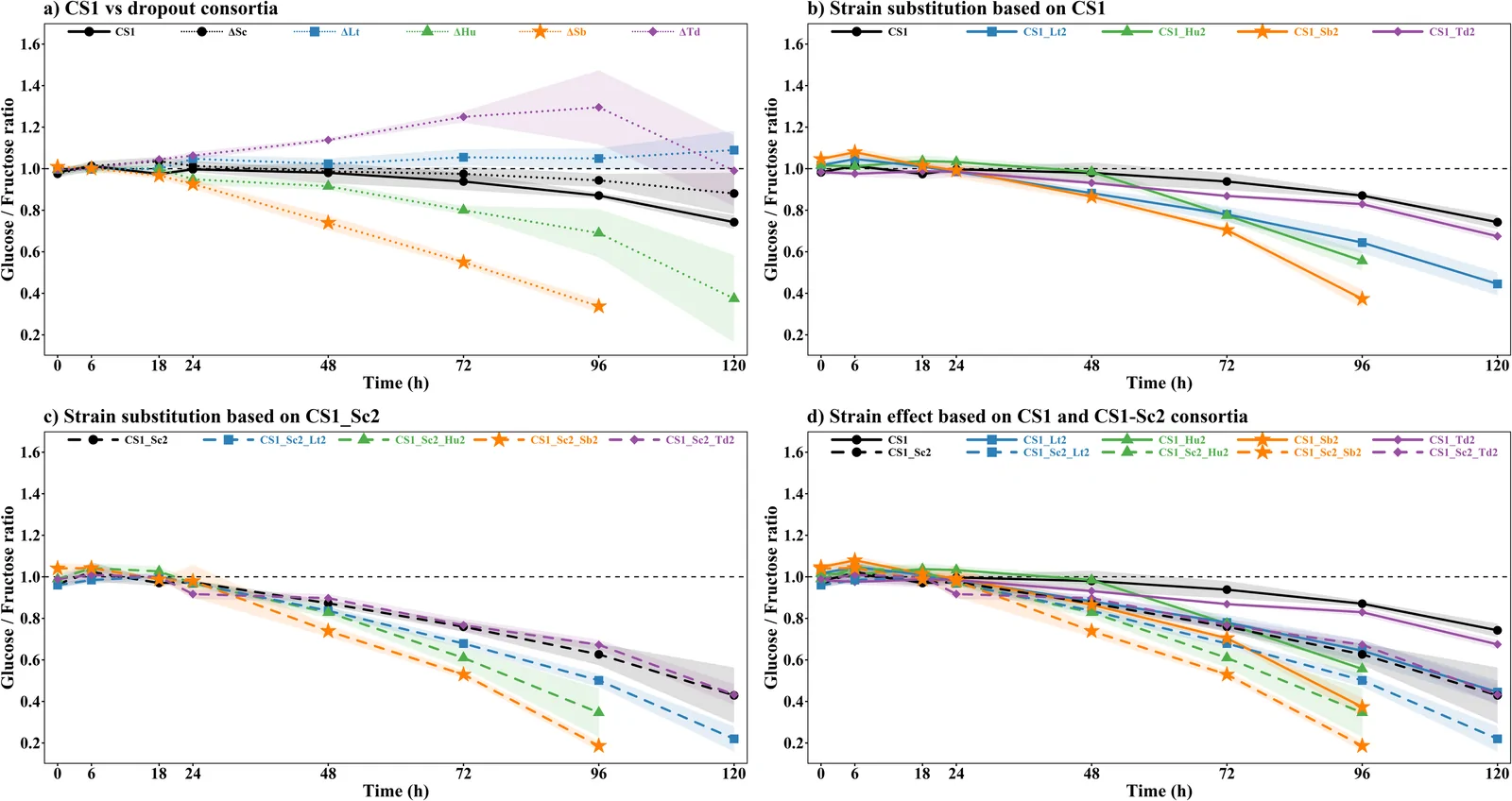
Sugar utilization across 15 synthetic yeast consortia. The glucose-to-fructose ratio (G/F ratio) was calculated at each sampling point from enzymatic quantification of residual glucose and fructose, and plotted over the fermentation time course. Curves represent the mean of three biological replicates; shaded bands denote ±SEM. (**a**) CS1 baseline compared with the five single-species drop-out consortia (ΔSc, ΔLt, ΔHu, ΔSb, and ΔTd). (**b**) Strain-switch consortia constructed on the Sc1 background (CS1_Lt2, CS1_Hu2, CS1_Sb2, and CS1_Td2), with CS1 shown for reference. (**c**) Strain-switch consortia constructed on the Sc2 background (CS1_Sc2_Lt2, CS1_Sc2_Hu2, CS1_Sc2_Sb2, and CS1_Sc2_Td2), with CS1_Sc2 shown for reference. (**d**) Pairwise comparison of Sc1 vs Sc2 backgrounds across matched consortia, isolating the effect of Sc strain identity. Colors follow the scheme used in Fig. 2. The dashed horizontal line at G/F = 1 indicates equimolar glucose and fructose consumption.

Sugar utilization was characterized by the temporal evolution of the glucose-to-fructose ratio (G/F ratio) across the 15 consortia from 0 to 120 h (Fig. 3). In grape must, glucose and fructose are present in approximately equimolar amounts, yet the yeast species active during fermentation differ markedly in their relative preference for the two hexoses, from the strongly glucophilic *S. cerevisiae* to fructophilic non-*Saccharomyces* species such as *S. bacillaris* (43). The glucose-to-fructose ratio therefore reflects the combined hexose preferences of the active community rather than the bulk rate of sugar depletion, and a shift in this ratio provides a functional readout of how the community’s carbon utilization is restructured when its composition changes. For this reason, rather than the rate of sugar consumption, the present analysis focused on sugar preference, that is, whether strain substitution or species drop-out shifted the relative utilization of glucose and fructose at the consortium level. Overall, the G/F ratio profiles differed across both the drop-out and the strain-substitution consortia. The CS1 consortium maintained a near-balanced pattern, with the G/F ratio close to one throughout most of the fermentation, shifting slightly toward a glucophilic profile in the late stage; the ΔSc consortium followed a similar trajectory, with the G/F ratio remaining closer to one (Fig. 3a). In contrast, the four remaining drop-out consortia shifted away from this near-balanced profile in two opposing directions: the absence of Lt or Td shifted the consortium toward more rapid fructose consumption, with the effect more pronounced in ΔTd, while absence of Hu or Sb shifted it toward more rapid glucose consumption (Fig. 3a). The strain-switch consortia exhibited distinct G/F ratio profiles depending on the substituted non-*Saccharomyces* strains. Under both Sc1 and Sc2 backgrounds, switching to Lt2, Hu2, or Sb2 shifted the consortium toward more rapid glucose consumption, whereas switching to Td2 produced a profile comparable to that of the corresponding reference consortium (Fig. 3b and c). Pairwise comparison of Sc1 vs Sc2 backgrounds across matched consortia revealed that Sc2-background consortia consistently consumed glucose more rapidly than their Sc1 counterparts (Fig. 3d). The raw glucose and fructose consumption curves are provided in Fig. S2; consortia with more rapid sugar consumption corresponded to faster cumulative CO_2_ release (Fig. 2a).

### Population succession revealed species- and strain-specific impacts on yeast population dynamics

**Fig 4:**
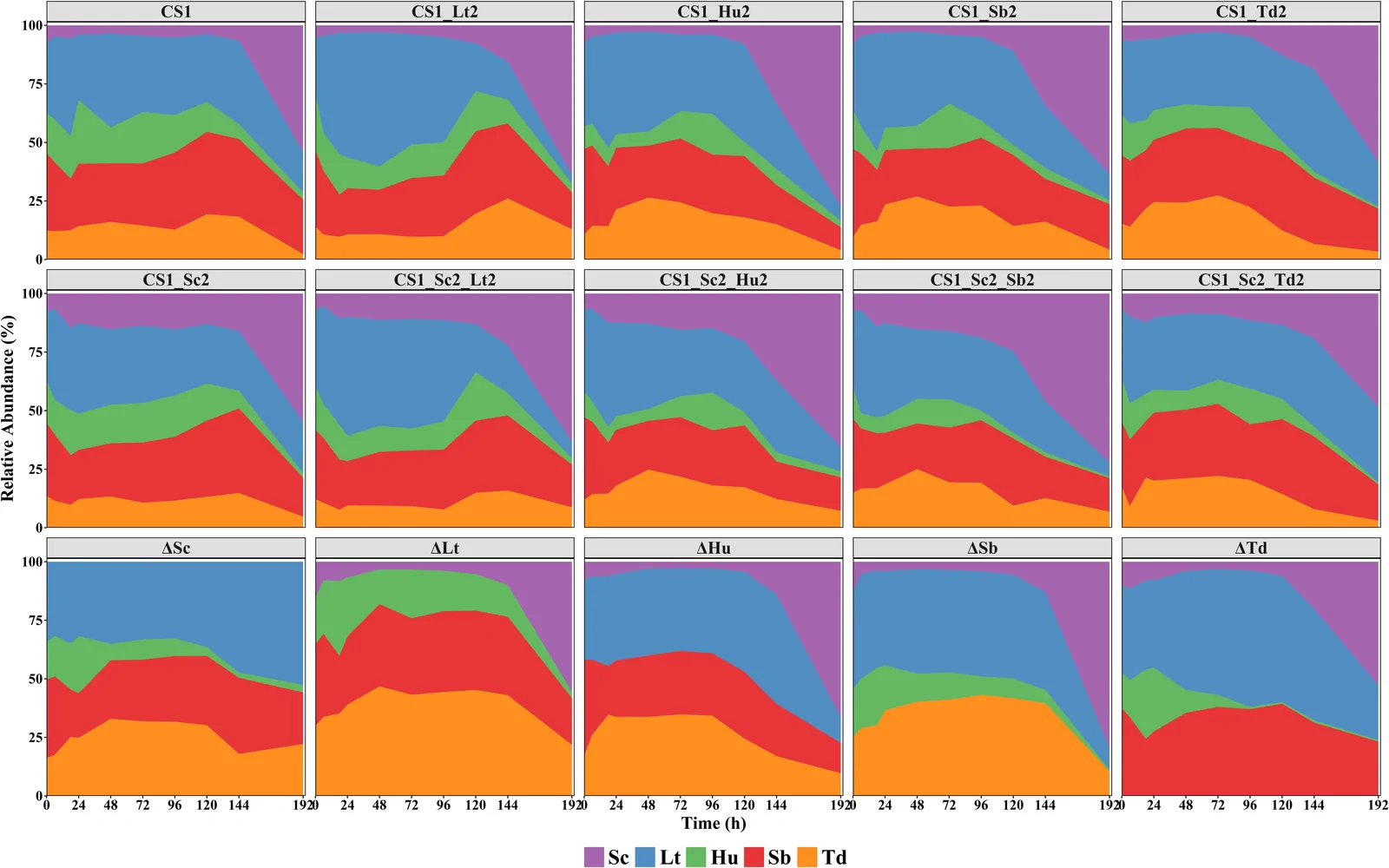
Stacked area plots depicting population dynamics of 15 synthetic yeast consortia. Relative abundance of each species was determined by ARISA at 10 sampling points across the 192-h fermentation. Each panel represents one consortium, with stacked colors indicating species identity (Sc, purple; Lt, blue; Hu, green; Sb, red; Td, orange). Rows organize consortia by experimental design: top row, CS1 baseline and strain-switch consortia on the Sc1 background (CS1, CS1_Lt2, CS1_Hu2, CS1_Sb2, and CS1_Td2); middle row, corresponding consortia on the Sc2 background (CS1_Sc2, CS1_Sc2_Lt2, CS1_Sc2_Hu2, CS1_Sc2_Sb2, and CS1_Sc2_Td2); bottom row, single-species drop-out consortia (ΔSc, ΔLt, ΔHu, ΔSb, and ΔTd). Curves represent the mean of three biological replicates.

Population dynamics across the 15 consortia were characterized by ARISA-based relative abundance of each species at 10 sampling points throughout the 192-h fermentation period (Fig. 4) and also through determination of the viable culturable population on differential media (Fig. S3). A common succession pattern was observed in all consortia, in which non-*Saccharomyces* species dominated the early and mid-fermentation, while Sc increased in relative abundance from approximately 120 h onward and became the most abundant species by the end of fermentation. Hu was the first to decline, at approximately 120 h, while Sb and Td maintained stable proportions; Lt occupied the largest relative abundance area and declined only in the late fermentation. Under the Sc1 background, switching Lt1 to Lt2 produced the most visible shift, with Lt2 occupying a higher relative abundance during the early fermentation stage and decreasing more steeply by the late stage; switching the other three non-*Saccharomyces* species produced more limited shifts (Fig. 4, top row). The same pattern of Lt2 strain effect was observed under the Sc2 background (Fig. 4, middle row). Comparison between the Sc1 and Sc2 backgrounds showed that Sc occupied a larger relative abundance area in the Sc2-background consortia than in their Sc1 counterparts. The drop-out consortia exhibited the largest abundance shifts among all configurations (Fig. 4, bottom row). In ΔLt and ΔSb, Td occupied a substantially larger relative abundance area throughout the fermentation, whereas in ΔTd, both Lt and Sb expanded into the abundance space otherwise occupied by Td. In ΔSc, Lt maintained a high relative abundance throughout the fermentation. In ΔHu, the remaining four species maintained relatively stable proportions until the late-stage rise of Sc.

### Consortia showed different primary metabolite profiles

Primary metabolites were quantified by high-performance liquid chromatography (HPLC) at the end of fermentation across the 15 consortia (Fig. S4), and the consortium-driven variation in each metabolite was assessed by analysis of variance (ANOVA; Fig. S5) and Dunnett’s test (Fig. S6b). By one-way ANOVA, each metabolite varied significantly (*q* < 0.05; Fig. S5A, column 1), except for citric acid (*q* = 0.075). The two-way ANOVA, fitted to the 10 substitution consortia, partitioned this variation into the effects of the *S. cerevisiae* strain and the non-*Saccharomyces* partner. Substituting the *S. cerevisiae* strain (Sc1 to Sc2) significantly affected lactic acid, acetic acid, and glycerol, but had little to no effect on the remaining metabolites (Fig. S5A, column 2). In contrast, substituting the non-*Saccharomyces* partner significantly affected every metabolite except citric acid (Fig. S5A, column 3), indicating that the non-*Saccharomyces* strains selected in this study exerted a broader impact on the primary metabolite output than the *S. cerevisiae* strains tested. To assess whether the effect of substituting Sc1 with Sc2 was consistent across community contexts or instead depended on the identity of the non-Saccharomyces partner, the interaction term of the two-way ANOVA (Fig. S5A, column 4) was examined together with the corresponding interaction plots (Fig. S6A). The effect was directionally uniform across all five backgrounds: lactic acid and glycerol consistently decreased, and acetic acid consistently increased from the Sc1 to the Sc2 background, with only the magnitude of the change varying between consortia. The absence of a significant interaction therefore indicates that, for these metabolites, the *S. cerevisiae* strain effect was largely independent of the non-*Saccharomyces* background. The remaining metabolites did not show a consistent directional response and were therefore not interpreted further.

Dunnett’s comparisons of each single-species drop-out consortium against the control community (CS1) were largely non-significant, consistent with the generally low concentrations of citric, malic, and succinic acids (Fig. S4 and S6b). The clearest exception was lactic acid, which fell markedly below CS1 in the consortium lacking *L. thermotolerans*. Also, a smaller but significant reduction was observed in the *S. bacillaris* drop-out (ΔSb).

**Fig 5:**
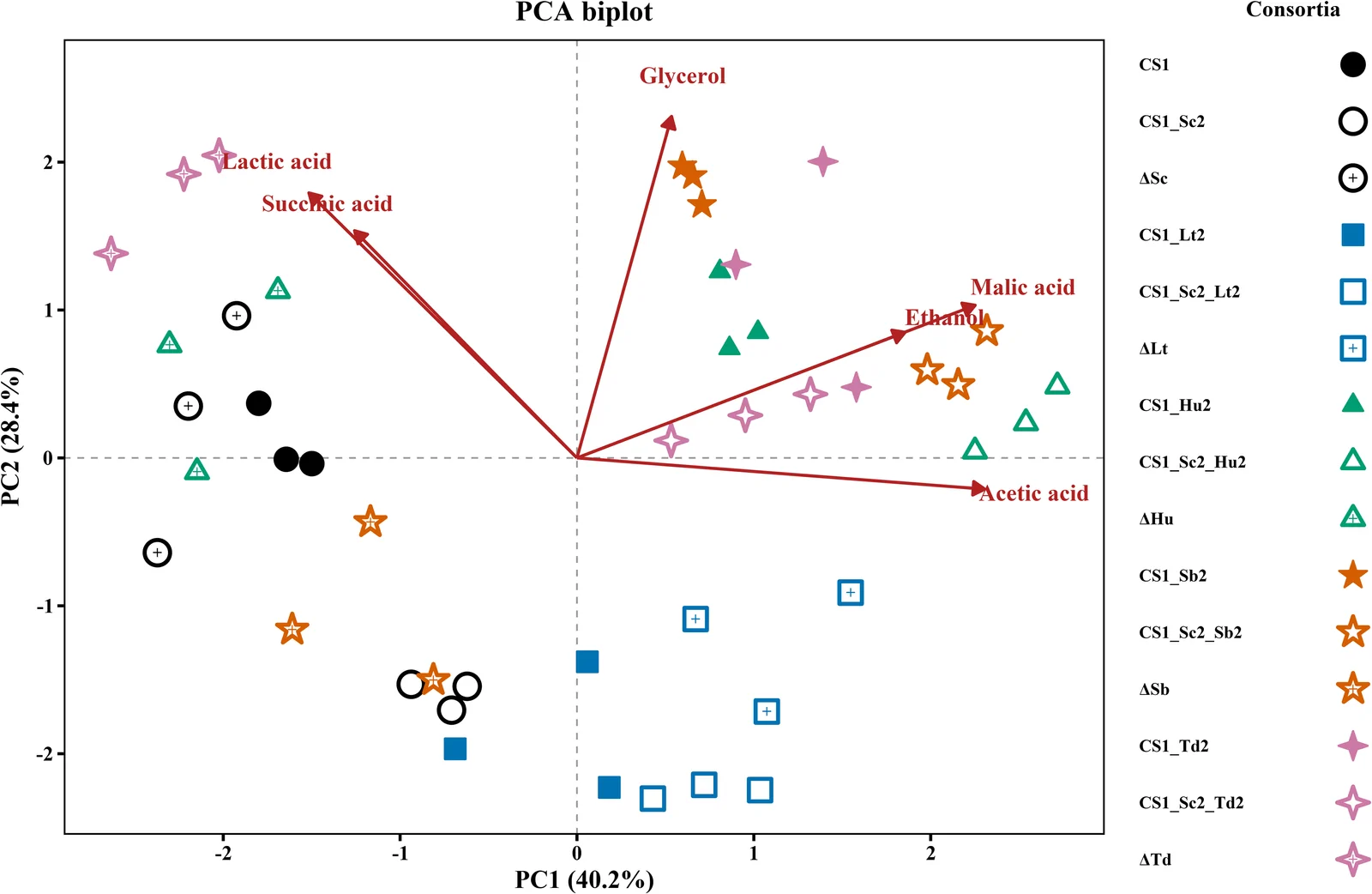
Principal component analysis bi-plot of six primary metabolite profiles across 15 synthetic yeast consortia at the end of fermentation. Citric acid was excluded due to its negligible variation across consortia. Each point represents one biological replicate, with three replicates shown per consortium. The first two principal components are shown, with the percentage of total variance explained indicated on each axis. Metabolite loading vectors are overlaid, with vector direction and length indicating each metabolite’s contribution to the ordination.

Principal component analysis of the six significantly varying metabolites (citric acid excluded) summarized the metabolic profiles of the fifteen consortia (Fig. 5). The first two components explained 68.6% of the variance (PC1, 40.2%; PC2, 28.4%), and the close grouping of replicates confirmed reproducible profiles. PC1, defined by acetic acid, malic acid, and ethanol, ordered the consortia by the extent of these fermentation products, whereas PC2, defined by glycerol and lactic acid, resolved the lactic acid signal contributed by *L. thermotolerans*. The distribution was governed primarily by the non-*Saccharomyces* species present. The consortia that showed reduced production in glycerol or lactic acid were positioned toward the low-glycerol/low-lactic-acid extreme of PC2. This group comprised the Lt2-containing consortia and ΔLt, in which lactic acid production was reduced, and ΔSb, in which loss of the glycerol-rich *S. bacillaris* lowered the glycerol signal. In contrast, ΔTd and the Sb2-containing consortia were clustered in the opposing extreme. Along PC1, consortia retaining an active Hu2, Sb2, or Td2 partner separated toward the acetic acid and ethanol vectors, whereas CS1 and the ΔSc and ΔHu drop-outs lay at the opposite extreme; ΔTd was extreme on both axes. Upon this partner-defined structure, *S. cerevisiae* substitution imposed only a secondary shift, the Sc2-containing consortia lying consistently further along positive PC1 than their Sc1 counterparts, which is a hierarchy that parallels the partition resolved by the two-way ANOVA.

### Volatile profiles differed across drop-out and strain-substitution consortia

Major volatiles were quantified by gas chromatography with flame ionization detection (GC-FID) at the end of fermentation across all 15 consortia, while only 15 compounds that were quantifiable are presented (Fig. S7), and the consortium-driven variation in each compound was assessed by ANOVA (Fig. S9) and Dunnett’s test (Fig. S10b). By one-way ANOVA, all 15 compounds varied significantly among the consortia (*q* < 0.05; Fig. S9a, column 1), although the variation in n-butanol and acetoin was less marked than that of the other compounds.

The two-way ANOVA, fitted to the 10 substitution consortia, partitioned this variation into the effects of the *S. cerevisiae* strain and the non-*Saccharomyces* partner. Substituting the non-*Saccharomyces* partner significantly affected all 15 compounds (Fig. S9a, column 3), whereas substituting the *S. cerevisiae* strain (Sc1 → Sc2) significantly affected only four (n-propanol, ethyl lactate, ethyl octanoate, and hexanoic acid; Fig. S9a, column 2). As for the primary metabolites, the non-*Saccharomyces* strains selected in this study therefore exerted a broader impact on the volatile output than the *S. cerevisiae* strains tested. For most compounds, no significant strain-by-partner interaction was detected, indicating that the effect of the *S. cerevisiae* strain was directionally consistent across the non-*Saccharomyces* backgrounds, while a significant interaction was found only for ethyl acetate, 3-ethoxy-1-propanol, isobutyric acid, and hexanoic acid (Fig. S9a, column 4 & Fig. S10a).

Dunnett’s comparisons of each single-species drop-out consortium against the control community (CS1) revealed mostly positive shifts relative to CS1, with many higher alcohols, fatty acids, and esters increasing upon removal of a non-*Saccharomyces* species (Fig. S10b). Ethyl lactate was the clearest directional exception: it fell markedly below CS1 in the consortium lacking *L. thermotolerans* (ΔLt), while increasing in the other drop-outs, consistent with *L. thermotolerans* being the principal source of its precursor.

**Fig 6:**
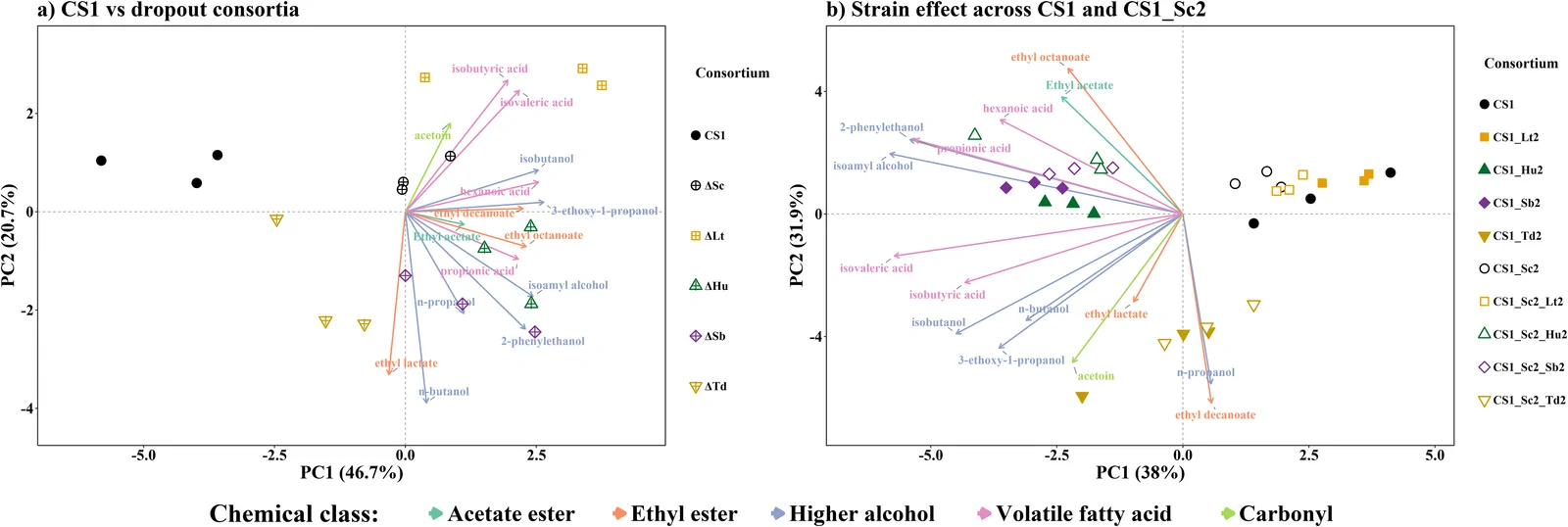
Principal component analysis bi-plots of 15 major volatile compounds measured by GC-FID at the end of fermentation across defined subsets of synthetic yeast consortia. Each PC plot presents a subset of consortia: (**a**) CS1 baseline compared with the five single-species drop-out consortia (ΔSc, ΔLt, ΔHu, ΔSb, and ΔTd). (**b**) All 10 strain-switch consortia under both Sc1 and Sc2 backgrounds. In each panel, each point represents one biological replicate, with three replicates shown per consortium; volatile loading vectors are overlaid. The percentage of total variance explained is indicated on each axis.

To examine the overall volatile structure, principal component analysis was performed. A global ordination of all 15 consortia is provided as an overview (Fig. S8), and two focused ordinations are shown in Fig. 6. The three biological replicates of each consortium grouped closely throughout, indicating reproducible volatile profiles.

The first ordination compared CS1 (the standard consortium) with the five drop-out consortia (Fig. 6a). CS1 lay away from most of the volatile loading vectors, consistent with its generally lower concentrations of these compounds. ΔLt separated from the other drop-out consortia along the ethyl lactate vector, mirroring its marked loss of ethyl lactate identified by Dunnett’s test, whereas ΔHu and ΔSb grouped together in association with several higher alcohols, fatty acids, and esters, and ΔTd separated along the higher-alcohol and fatty-acid vectors.

The second ordination incorporated all 10 substitution consortia under both the Sc1 and Sc2 backgrounds (Fig. 6b). The consortia grouped primarily according to the non-*Saccharomyces* substitution, and the Sc1- and Sc2-background consortia were not separated into distinct regions of the ordination. Without using any grouping information, this arrangement independently recovered the structure resolved by the ANOVA, in which the non-*Saccharomyces* partners, rather than the *S. cerevisiae* strains, were the broader source of variation.

## DISCUSSION

The present study examined how inter- and intra-species interactions contribute to the behavior of a defined multi-species wine yeast consortium. Here, a five-species consortium was constructed to provide a tractable system while retaining the ecological complexity of a wine-ecosystem relevant community. Following this design, two levels of perturbation were applied: species drop-out, which tests whether the absence of a species has a significant impact, and strain substitution, which tests whether strain identity matters. Across five phenotypic dimensions—fermentation kinetics, sugar utilization, population dynamics, primary metabolites, and volatile metabolite profiles—the two levels reshaped consortium behavior in distinct and dimension-specific ways: (i) species drop-out produced significant and directional shifts, most pronounced in the growth-related dimensions, and (ii) strain substitution produced smaller but reproducible and significant shifts that were most evident in the metabolic output. This pattern is consistent with an interpretation in which species identity establishes the baseline functional structure of the consortium while strain identity modulates the magnitude of that structure. The finding extends the work of Bagheri et al. (4), who demonstrated that ecological interactions, rather than physico-chemical conditions alone, govern the contribution of individual species to fermentation; the present data add a finer layer of resolution by showing that intraspecific variation also propagates measurably to the community level. Such complex outcomes produced from ecological interactions are not unique to winemaking, nor limited to yeast-yeast interactions. Across fermented-food ecosystems, microbial interactions rather than abiotic conditions alone shape the community structure and function (49, 50). Cross-domain communities such as cheese (51), sourdough (52), and kombucha (53) have shown the significance of studying their complex microbial ecological interactions and the effects of individual species and community-level outputs. Therefore, understanding such ecological interactions is pivotal to explaining, and further controlling, the functional output of any microbial community: a question this study addresses at the level of a defined yeast consortium in winemaking.

The drop-out series provided the clearest evidence of species-level effects. The absence of *L. thermotolerans* markedly prolonged the lag phase, indicating a strong role for this species in the early adaptation of the community to the medium (37), and caused the near-complete loss of lactic acid and its downstream ester ethyl lactate, consistent with this species being the principal and effectively sole source of this metabolic route. The absence of *S. cerevisiae* lowered the final cumulative CO_2_ yield (Fig. 2), yet the sugars were exhausted (<0.5 g L^−1^), indicating that carbon was redirected away from CO_2_/ethanol. As no significant increase was observed in glycerol or any other measured primary metabolites, the most parsimonious explanation is a shift of carbon toward biomass, although biomass was not quantified in this study. Notably, for metabolites typically attributed to a single producer species, removal of that species reduced the corresponding output only modestly at the consortium level: glycerol yield declined when the strongly glycerogenic *S. bacillaris* was removed, and ethanol yield declined in the absence of *S. cerevisiae*, yet in neither case was the reduction pronounced. This limited response suggests that the production of these metabolites is a community-level outcome to which several members contribute.

The sugar utilization and population dynamics data, read together, further revealed interactions among the non-*Saccharomyces* species, principally among *L. thermotolerans*, *S. bacillaris*, and *T. delbrueckii*. Given that *S. bacillaris* is strongly fructophilic (43), its removal shifted the consortium toward more rapid glucose consumption, whereas the removal of *T. delbrueckii* shifted it significantly toward fructose consumption. These shifts corresponded to compensatory changes in population structure: *T. delbrueckii* expanded in the ΔSb and ΔLt consortia, while both *L. thermotolerans* and *S. bacillaris* expanded in ΔTd, occupying the abundance space otherwise held by *T. delbrueckii*. Together with the reported suppressive effect of *T. delbrueckii* on *L. thermotolerans* (54), these observations point to mutual competition among the non-*Saccharomyces* species. This is notable because interactions between *Saccharomyces* and non-*Saccharomyces* species are well documented, whereas direct evidence for mutual interactions among non-*Saccharomyces* species is comparatively scarce (20). The drop-out experiments thus indicate that species-level interactions are a principal determinant of consortium behavior, in agreement with the central hypothesis of this study. Moreover, four species used in this study (*S. cerevisiae*, *L. thermotolerans*, *H. uvarum*, and *T. delbrueckii*) are known producers of killer toxins (55–58), and *S. bacillaris* has been reported to carry killer toxin genes (59). In the consortium, all species remained culturable until the end of fermentation, except for *H. uvarum*, which declined earlier, consistent with its well-documented intolerance of high ethanol concentrations (60). Assessing the ecological contribution of such secreted toxins in a community context would be of considerable interest but would require omics-level investigations.

Besides the assessment of sugar consumptions, direct physical cell-to-cell contact has been shown to elicit species-specific responses at the transcriptional level, both in binary systems (*S. cerevisiae* and *L. thermotolerans*) (22, 61) and in a three-species system (*S. cerevisiae*, *L. thermotolerans*, and *T. delbrueckii*) (54). Such data are currently under investigation for this five-species system and will provide leads for causal molecular interaction mechanisms in more complex ecosystems. Furthermore, differences in nitrogen and amino acid preferences, characterized mainly in monocultures or co-culture systems (62–65), suggest that individual nitrogen preferences may also impact the population dynamics observed in this study. However, to our knowledge, such different nitrogen and amino acid preferences have not been examined in a multispecies context. The present system resolves the consequences of species and strain identity at the phenotypic level and provides a reproducible system in which ecological mechanisms and environmental factors in a multi-species consortium could be dissected in future work.

Among the metabolites quantified, lactic acid offered a uniquely tractable readout of these effects, because within this consortium it is produced predominantly by a single member, *L. thermotolerans*, with Lt1 in this study being the only strain showing high activity of the lactic acid pathway (48). This single-compound resolution revealed two distinct layers of variation. First, lactic acid production depended on the Lt strain present, falling sharply when the producing strain Lt1 was absent (ΔLt) or replaced by the non-producing strain Lt2. Second, and more notably, among the consortia that all retained Lt1, lactic acid concentration still varied with the substitution of other non-*Saccharomyces* strains. Since the producing strain itself was unchanged, this variation indicates that the metabolic output of one species can be modulated by the identity of its community partners (65–68). Ethyl lactate, the ester derived directly from lactic acid, tracked the same pattern in the volatile data set (Fig. 6; Fig. S7). This correspondence, observed independently by HPLC and GC, supports the interpretation that the signal reflects a genuine metabolic phenomenon rather than an analytical artifact and establishes lactic acid as a near-ideal tracer of inter-species interaction within this system. However, such single-species attribution is the exception rather than the rule. Metabolites conventionally marked as species characteristic, such as high acetic acid yield in *H. uvarum* and glycerol in *S. bacillaris*, are produced by multiple members within a consortium context. Acetic acid is generated broadly through oxidative and stress-related metabolism across species (69), and consistent with this, the absence of either *S. cerevisiae* or *H. uvarum* lowered acetic acid production, although not significantly compared to the control consortium (CS1; Fig. S4). Glycerol, likewise, is produced by all fermenting yeasts as part of redox balancing; although its concentration varied significantly across consortia, the absence of *S. bacillaris* produced only a slight reduction relative to CS1, so that no single contributor could be resolved. The more informative signal is therefore not which species produces them, but how their concentrations varied across consortia differing in species and strain composition. Together with the interpretation from the principal component analysis (PCA) in HPLC and GC data (Fig. 5 and 6), the metabolic output becomes a readout that represents the ecological complexity of the community interactions rather than a marker of individual species activity.

Strain substitution produced effects that were moderate across the growth-related dimensions but more pronounced in the metabolic output, and their breadth differed between the non-*Saccharomyces* species and *S. cerevisiae*. Substitution of the non-*Saccharomyces* strains significantly affected the metabolite output, as resolved by the two-way analysis and PCA, and shifted sugar preference in a reproducible and systematic manner; because the substituted strains belong to the same species, these shifts represented a deviation in degree rather than in kind. In the population dynamics, no large strain-associated changes were apparent apart from Lt2, which occupied a higher relative abundance during the early fermentation. For *S. cerevisiae*, because every non-*Saccharomyces* substitution was tested under both Sc backgrounds, the two Sc strains could be compared directly, and they produced a real but limited strain effect. In sugar utilization, the Sc2-background consortia consumed glucose more rapidly than their Sc1 counterparts, in line with the greater fructose-utilizing capacity of the Sc1 strain. In the metabolic output, the two-way analysis resolved a significant main effect of the *S. cerevisiae* strain for a small subset of compounds, confirming that switching Sc1 to Sc2 altered the final metabolite profile, albeit across fewer compounds than the non-*Saccharomyces* substitution did. The differentiation attributable to the *S. cerevisiae* strain was therefore present but smaller than that attributable to the non-*Saccharomyces* strains.

One observation worth highlighting is that *S. cerevisiae* strains naturally present or inoculated at low initial density typically rise to dominance when nutrients are exhausted and ethanol accumulates, a pattern mirroring the late emergence of *S. cerevisiae* in spontaneous wine fermentation (70–73) and is reproduced in the present system. Here, *S. cerevisiae* was inoculated at the lowest initial density and grew slowly through the early and middle stages; its dominance rose only from approximately 120 h, when residual total sugars fell to around or less than 20 g L⁻¹ (Fig. S2), and non-*Saccharomyces* species declined (Fig. S3). Importantly, low relative abundance does not equate to metabolic silence. Despite their low relative abundance in persistence during fermentation, both *S. cerevisiae* strains ultimately contributed a detectable metabolic footprint in primary metabolites (Fig. 5; Fig. S4 and S6) and major volatiles (Fig. 6; Fig. S7 and S10). Its footprint is therefore better described as smaller than that of the non-*Saccharomyces* partners rather than negligible (Fig. S5 and S9). This late-dominant, low-footprint pattern offers an ecological rationale for a long-standing but hitherto anecdotal observation among practitioners of spontaneous fermentation, namely that the specific *S. cerevisiae* population is a less decisive determinant of the fermentation outcome than is commonly assumed. The present data provide, to our knowledge, a first systematic and controlled line of evidence consistent with that view, and suggest that the metabolic identity of a spontaneous wine is shaped from yeast ecological interactions during the early, non-*Saccharomyces*-dominated phase of fermentation.

The present study is based primarily on descriptive, phenotypic observations, and the close grouping of biological replicates throughout the multivariate analyses confirms that the consortium behaves reproducibly. Such reproducibility is a prerequisite for resolving effects as fine as single-strain substitution. The use of a chemically defined synthetic must enabled this reproducibility but constrains direct extrapolation to industrial fermentation; it is therefore the relative patterns reported here, rather than the absolute values, that are expected to be the more transferable outcome. The three biological replicates per consortium also limit the power to detect interaction effects, so that a non-significant interaction should be read as the absence of a detectable interaction rather than its definitive absence. This study was designed as a phenotypic-level characterization, and the molecular mechanisms underlying the observed species- and strain-dependent effects were not addressed in the present work. Mechanisms that were studied to mediate yeast-yeast interactions, including direct cell-to-cell contact and/or different preferences in nitrogen and amino acid utilization, could significantly contribute to the patterns reported in this study, and resolving their relative importance will require targeted approaches in a multi-species community. Despite this limitation, this study provides a systematic, multi-dimensional account of how species and strain identity jointly shape consortium behavior, supporting a hierarchical interpretation in which species identity establishes the functional structure of the consortium and strain identity modulates its magnitude. A central question left open is the molecular basis of the strain-level effects observed here, the resolution of which will require approaches capable of linking genotype to community-level phenotype and represents the direction for subsequent work.

## MATERIALS AND METHODS

### Culture conditions

The yeast strains used in this study are listed in Table 1. The yeast consortium was established by five species, each represented by two strains of diverging phenotype. Species were selected for their representative association with wine fermentation, and strains were selected primarily based on their growth and fermentation kinetics. All strains were preserved in the culture collection center of the SAGWRI, Stellenbosch University, South Africa. All yeast stocks were maintained in 15% (vol/vol) glycerol at −80°C and were recovered on Wallerstein Laboratory Nutrient (WLN) agar (Sigma-Aldrich, South Africa) when required.

**TABLE 1:** Species and strains used in this study

Strains	Strain number	Source	Codes
*Saccharomyces cerevisiae*	PINNACLE	AB Biotek, UK	Sc1
*Saccharomyces cerevisiae*	Y1464	SAGWRI, Stellenbosch	Sc2
*Lachancea thermotolerans*	LEVEL^2^ LAKTIA	Lallemand, Canada	Lt1
*Lachancea thermotolerans*	VINIFLORA CONCERTO	Novonesis, Denmark	Lt2
*Hanseniaspora uvarum*	Y861	SAGWRI, Stellenbosch	Hu1
*Hanseniaspora uvarum*	Y966	SAGWRI, Stellenbosch	Hu2
*Starmerella bacillaris*	Y943	SAGWRI, Stellenbosch	Sb1
*Starmerella bacillaris*	Y547	Stark Conde, Stellenbosch	Sb2
*Torulaspora delbrueckii*	NJ783	SAGWRI, Stellenbosch	Td1
*Torulaspora delbrueckii*	LEVEL^2^ BIODIVA	Lallemand, Canada	Td2

### Micro-fermentation

All fermentations were conducted in synthetic grape must (SGM) to ensure reproducibility and to eliminate vintage-related variability. The SGM was prepared according to the formulation of Henschke and Jiranek (74) and Bely et al. (75), and the fermentation procedure followed that previously described by Bagheri et al. (28). Briefly, the SGM contained 200 g L^−1^ total sugars (glucose and fructose at 100 g L^−1^ each), 300 mg L^−1^ assimilable nitrogen (460 mg L^−1^ NH₄Cl and 180 mg L^−1^ amino acids), and was supplemented with organic acids, vitamins, trace elements, and anaerobic factors required for yeast growth. Fermentations were conducted in 150 mL SGM dispensed in 250 mL Erlenmeyer flasks at 25°C with agitation (110 rpm) to ensure homogeneity.

Inoculation densities were optimized to ensure that all five species were able to metabolically and actively contribute to the fermentation (Table S1). *S. cerevisiae* was inoculated at a lower density (2 × 10^5^ cells mL^−1^) to prevent the early competitive exclusion of non-*Saccharomyces* species. *H. uvarum* was inoculated at 5 × 10^5^ cells mL^−1^, as higher initial densities were found in preliminary trials to suppress co-inoculated species, consistent with its characteristically rapid proliferation. The remaining three species (*L. thermotolerans*, *S. bacillaris*, and *T. delbrueckii*) were each inoculated at 1 × 10^6^ cells mL^−1^. Inoculum concentrations were determined from calibration curves relating OD₆₀₀ to cell density, with cell enumeration performed by flow cytometry (Table S2).

Cumulative CO_2_ release was monitored gravimetrically at 10 sampling time points (0, 6, 18, 24, 48, 72, 96, 120, 144, and 192 h). Four descriptive kinetic parameters were extracted from each replicate’s CO_2_ release curve by linear interpolation between adjacent sampling points, without model fitting or curve smoothing. The lag phase was defined as the time required to reach 5 g L^−1^ of cumulative CO_2_. The maximum CO_2_ release rate (V_max) was defined as the largest mean rate between any two adjacent sampling points. The time to 50% of total CO_2_ release (t_50_) was defined as the time required to reach half of the replicate’s maximum cumulative CO_2_ release. The maximum cumulative CO_2_ release was defined as the highest value recorded over the 192 h time course.

These parameters were used as comparative descriptors for cross-consortium analysis rather than as quantitative estimates of microbial physiology. Given the sampling resolution, absolute values, particularly the lag phase, should not be interpreted as precise physiological parameters of the underlying yeast populations, but rather as standardized descriptors enabling the parallel comparison of kinetic profiles across all consortia.

### Population dynamics

Population dynamics throughout fermentation were monitored primarily by Automated Ribosomal Intergenic Spacer Analysis (ARISA), a molecular method for tracking species composition in this wine yeast consortium that followed a similar approach to Bagheri et al. (29). ARISA was selected as the primary tool due to the ease of manipulation of large quantities of samples, minimizing run-to-run variability during analysis. However, ARISA does not distinguish between metabolically active and non-viable cells (76); its readout is therefore interpreted as succession patterns compared across consortia rather than as absolute viable population sizes. Enumeration of viable culturable cells by direct plating on WLN agar was performed as a complementary assessment. Samples for both methods were withdrawn at the 10 sampling time points used for CO_2_ monitoring (see section Micro-fermentation).

ARISA resolves community composition on the basis of the species-specific length heterogeneity of the ITS1–5.8S rRNA–ITS2 region. Genomic DNA was extracted from fermentation samples following the method of Sambrook and Russell (77). The ITS region was amplified using the carboxy-fluorescein-labeled ITS1 primer (5′−6-FAM-TCC GTA GGT GAA CCT TGC GG-3′) and the ITS4 primer (5′-TCC TCC GCT TAT TGA TAT GC-3′). Amplified fragments were separated by capillary electrophoresis on an ABI 3100 Genetic Analyzer (Applied Biosystems) with a LIZ1200 size standard, and profiles were analyzed using GeneMapper software version 4.1 (Applied Biosystems). Only peaks with an area exceeding 0.5% of the total fluorescence were retained, and bin sizes of 3 bp (for ITS regions below 700 bp: *S. bacillaris* and *L. thermotolerans*) and 5 bp (for ITS regions above 700 bp: *H. uvarum*, *T. delbrueckii*, and *S. cerevisiae*) were applied to minimize analytical inaccuracies (78). The relative abundance of each species was calculated as the proportion of its peak area relative to the total peak area.

For viable cell enumeration, serial dilutions of fermentation samples were plated onto WLN agar, incubated at 30°C for 3 days, and species were differentiated based on their distinct colony morphology.

### Chemical analysis

#### Sugar quantification

Throughout fermentation, glucose and fructose concentrations were monitored enzymatically using commercial kits (D-glucose E5140 and D-fructose E5120; Roche Diagnostics, R-Biopharm, Darmstadt, Germany) on an Arena 20 XT automated analyzer (Thermo Fisher Scientific, Waltham, MA, USA) at the Chemical Analytical Laboratory of SAGWRI, Stellenbosch University. Fermentation was considered complete when the total residual sugar concentration fell below 2 g L^−1^. The enzymatic assay operated within a defined linear range, and its accuracy declined at low sugar concentrations; values below approximately 0.5 g L^−1^ exceeded the lower limit of the calibration range and were excluded from subsequent calculations. As residual sugar concentrations declined markedly in the late fermentation stage, the glucose-to-fructose ratio was calculated only up to 120 h, beyond which the low residual concentrations (each sugar below 2 g L^−1^) no longer permitted a reliable determination of sugar preference. To confirm that fermentation had reached completion despite the limited reliability of the enzymatic assay at low concentrations, final residual sugars were independently quantified by HPLC for all consortia; glucose was not detected, and fructose remained below 0.5 g L^−1^ in all samples.

#### Primary metabolite analysis

A high-performance liquid chromatography system (1260 Infinity, Agilent Technologies, Santa Clara, CA, USA) was employed to quantify key organic acids (malic, citric, succinic, lactic, and acetic acids), glycerol, and ethanol. Separation was performed on a Hi-Plex H column (300 × 7.7 mm, Agilent Technologies) maintained at 65°C, with isocratic elution using 5 mM H_2_SO_4_ at 0.6 mL min^−1^ and refractive index detection. Prior to analysis, samples were filtered through 0.45 μm syringe filters. Quantification was based on external calibration with certified reference standards (Sigma-Aldrich, St. Louis, MO, USA), using peak area measurements.

#### Major volatiles analysis

For volatile compound profiling, a liquid-liquid extraction protocol coupled with gas chromatography-flame ionization detection was implemented, following the methodology of Louw et al. (79x) with modifications. Fermentation samples (5 mL) received 4-methyl-2-pentanol internal standard (5 mg/L final concentration) before extraction with diethyl ether (1 mL). After sonication (5 min) and centrifugation (4,000 × *g*, 5 min), the upper organic layer was recovered and dehydrated with anhydrous Na_2_SO_4_. Analysis employed a Hewlett Packard 6890 Plus gas chromatograph (Agilent Technologies) fitted with a polar DB-FFAP capillary column (dimensions: 60 m length, 0.32 mm internal diameter, and 0.5 μm film thickness; Agilent Technologies). Hydrogen served as carrier gas (3.3 mL/min flow). The temperature gradient began at 33°C (17 min isothermal), increased at 12°C/min to 240°C, and then maintained at 240°C for 5 min. Split injection (ratio 15:1) was performed at 200°C with 3 μL sample volume, while the FID operated at 250°C. Compound identification utilized retention time matching against authentic standards, with quantification achieved via the internal standard approach.

#### Statistical analysis

All fermentations and chemical analyses were conducted in biological triplicates, and data are presented as the mean ± standard deviation. All statistical analyses and visualizations were performed in R (version 4.5.3; R Core Team) (80) with the *ggplot2* package (81). For each compound, differences among the 15 consortia were assessed by one-way ANOVA, followed by Tukey’s HSD post-hoc test where significant. To partition the sources of variation, a two-way ANOVA was fitted to the ten substitution consortia (a balanced 2 × 5 design) for the main effects of the *S. cerevisiae* strain and the non-*Saccharomyces* partner and their interaction, and each drop-out consortium was compared with the complete community (CS1) by Dunnett’s test (*multcomp* package [82]). Within each test family, *P*-values were adjusted across compounds by the Benjamini–Hochberg false discovery rate procedure (83), and significance was evaluated at *q* < 0.05. Principal component analysis (*prcomp*) was applied to the centered and scaled primary metabolite and major volatile data sets to examine compositional patterns among consortia; citric acid was excluded from the primary metabolite PCA owing to its lack of significant variation.

## Supplementary Material

Reviewer comments

## Data Availability

This study did not generate nucleotide or amino acid sequences, structural data, or mass spectrometry-based omics datasets requiring deposition in a specialized repository. All data supporting the conclusions of this study are included in the article and its supplemental material. The yeast strains used are listed in Table 1, together with their culture collection identifiers and sources; wild-isolated strains held in the SAGWRI culture collection are available from the corresponding author upon reasonable request.

## SUPPLEMENTAL MATERIAL

Supplemental figures and tables spectrum.02819-26-s0001.pdf Fig. S1 to S10, and Tables S1 to S2.

OPEN PEER REVIEW reviewer-comments.pdf An accounting of the reviewer comments and feedback.

ASM does not own the copyrights to Supplemental Material that may be linked to, or accessed through, an article. The authors have granted ASM a non-exclusive, world-wide license to publish the Supplemental Material files. Please contact the corresponding author directly for reuse.
