# Supplementary material for: Species and strain-specific phenotypes shape performance and output of a five-species yeast consortium
Source: Microbiol Spectr. 2026 Sep 24;14(11):e02819-26. doi: 10.1128/spectrum.02819-26 (PMC13729150; doi:10.1128/spectrum.02819-26)
Supplement: Supplementary file 1 [file reviewer-comments.pdf]

ASM Microbiology Spectrum

Species and strain-specific phenotypes shape performance and output of a five-species yeast consortium

Fanzhi Wang, Jeanne Laforge, Mathabatha Setati, and Florian Bauer

Corresponding Author(s): Florian Bauer, Stellenbosch University

Review Timeline:

Submission Date:	September 1, 2026
Editorial Decision:	September 4, 2026
Revision Received:	September 14, 2026
Accepted:	September 14, 2026

Editor: Blaire Steven

Reviewer(s): The reviewers have opted to remain anonymous.

Transaction Report:

(Note: With the exception of the correction of typographical or spelling errors that could be a source of ambiguity, letters and reports are not edited. The original formatting of letters and referee reports may not be reflected in this compilation.)

DOI: https://doi.org/10.1128/spectrum.02819-26

Re: Spectrum02819-26 (Species- and strain-specific phenotypes shape yeast ecosystems)

Dear Prof. Florian F Bauer:

Thank you for the privilege of reviewing your work. Below you will find my comments, instructions from the Spectrum editorial office, and the reviewer comments.

After having read the author's response to previous review and the manuscript I feel that the authors have sufficiently addressed the reviewers concerns. As the senior editor though I still have one concert with the manuscript. I feel that the title is far too general and does not adequately address the experiment system or relevant species. It sounds almost more like the title of a review article. I would be happy to consider a revised manuscript with an expanded title.

Please return the manuscript within 60 days; if you cannot complete the modification within this time period, please contact me. If you do not wish to modify the manuscript and prefer to submit it to another journal, notify me immediately so that the manuscript may be formally withdrawn from consideration by Spectrum.

Revision Guidelines
To submit your modified manuscript, log into the submission site at https://spectrum.msubmit.net/cgi-bin/main.plex. Go to Author Tasks and click the appropriate manuscript title to begin. The information you entered when you first submitted the paper will be displayed; update this as necessary. Note the following requirements:

• Upload point-by-point responses to the issues raised by the reviewers in a file named "Response to Reviewers," NOT in your cover letter.
• Upload a compare copy of the manuscript (without figures) as a "Marked-Up Manuscript" file.
• Upload a clean .DOC/.DOCX version of the revised manuscript and remove the previous version.
• Each figure must be uploaded as a separate, editable, high-resolution file (TIFF or EPS preferred), and any multipanel figures must be assembled into one file.
• Any supplemental material intended for posting by ASM should be uploaded with their legends separate from the main manuscript. You can combine all supplemental material into one file (preferred) or split it into a maximum of 10 files with all associated legends included.

For complete guidelines on revision requirements, see our Submission and Review Process webpage. Submission of a paper that does not conform to guidelines may delay acceptance of your manuscript.

Data availability: ASM policy requires that data be available to the public upon online posting of the article, so please verify all links to sequence records, if present, and make sure that each number retrieves the full record of the data. If a new accession number is not linked or a link is broken, provide Spectrum production staff with the correct URL for the record. If the accession numbers for new data are not publicly accessible before the expected online posting of the article, publication may be delayed; please contact production staff (Spectrum@asmusa.org) immediately with the expected release date.

Publication Fees: For information on publication fees and which article types are subject to charges, visit ourwebsite. If your manuscript is accepted for publication and any fees apply, you will be contacted separately about payment during the production process; please follow the instructions in that e-mail. Arrangements for payment must be made before your article is published.

ASM Membership: Corresponding authors may join or renew ASM membership to obtain discounts on publication fees. Need to upgrade your membership level? Please contact Customer Service at Service@asmusa.org.

The ASM Journals program strives for constant improvement in our submission and publication process. Please tell us how we can improve your experience by taking this quick Author Survey.

Thank you for submitting your paper to Spectrum.

Sincerely,
Blaire Steven
Editor
Microbiology Spectrum

Response to Reviewers

Manuscript Spectrum02819-26

"Species- and strain-specific phenotypes shape yeast ecosystems"

We thank the Editor and both reviewers for their careful and constructive comments. Where a comment identified a genuine gap we revised the manuscript accordingly; for a small number of points we respectfully explain why the suggested change lies outside the scope and central question of this study. Reviewer comments appear on the left and our responses on the right.

**Reviewer 1**	**Response**
General: The manuscript by Wang and collaborators investigates the interactions established during synthetic wine must fermentation within 5 different wine yeasts, represented by two strains each, with different phenotypic characteristics.<br>The subject investigated is actual and relevant considering that the use of non-*Saccharomyces* yeasts have become common alternatives to *S. cerevisiae* monocultures starter cultures. Reading the title and the abstract I was expecting to read a bit more that a very descriptive paper in which the outcomes are reported in detail, however no insight into the mechanisms are not really reported.	We thank the reviewer for recognizing the relevance of the topic. This study is, by design, a systematic phenotypic characterization and we acknowledge the present study is limited in mechanistic dissection. The focus was to establish, in a defined multi-species consortium, whether species identity and strain identity make separable and reproducible contributions to community structure and function, a question that must be resolved at the phenotypic level before targeted mechanistic work can be meaningfully designed. The mechanistic layer is the explicit subject of planned follow-up work, and we have clarified this scope in the last paragraph of Discussion.
the selection of the non-Sacch species and strains was based almost only on fermentation kinetics (consumption of sugars), while it is well reported that the species selected can have different features that make them unique for the wine fermentations. I wander if *H. uvarum* strains produced different amounts of acetic acid, if *L. thermotollerans* has the capability to metabolize differently sugars for lactic acid production and *S. bacillaris* was able to produce different concentrations of glycerol. It would be beneficial for the reader to know about it.	Thank you. We would respectfully note that the strain- and species-selection rationale is already stated in the last paragraph of introduction and the first paragraph of the Results. And you are absolutely correct that it would be beneficial for the reader to know about every unique phenotypical trait of each strain. However, fermentation kinetics was used as a basic, deliberately chosen primary index, because our objective was to assemble pairs of conspecific strains that are phenotypically divergent in a defined, tractable way — not to pre-characterize every species-specific trait, which would have expanded the upstream work without serving the central question. We would also draw an important conceptual distinction: a metabolite can be attributed to a single species (as we discussed on lactic acid and *L. thermotolerans*) only when it derives essentially from one producer. Traits such as acetic acid or glycerol arise from multiple community members, and their consortium-level concentrations cannot be equated with the output of any one species in monoculture — community behavior and single-strain behavior are distinct by definition. Some words on the species-characteristic contributions the reviewer mentions (glycerol in absence of *S. bacillaris*) are in fact noted in the manuscript (Discussion 2nd paragraph); However, we have now made these points more prominent in the 4th paragraph of discussion to ensure they are not overlooked, and highlight that they are interpreted at the community level.

Reviewer 1	Response
the coinoculation was selected as a procedure to use, however with all the species selected in the study, the protocol that is suggested by yeast producing companies is almost all the time a sequential fermentation, where non Sacch are inoculated first and *S. cerevisiae* comes later (24- or 48-hours delay). I'm wondering how the change in the inoculation protocol could modify the interactions, since it has been already reported that it is for sure affecting the fermentation kinetics.	We thank the reviewer for raising this. We would clarify that co-inoculation was a direct consequence of the biological question, not an incidental choice. Our aim was to reproducibly characterize interactions that arise when community members are simultaneously present and competing from the outset, which most closely approximates the early phase of a spontaneous fermentation in nature, in which resident species co-occur rather than being introduced in a staggered, human-imposed sequence. The reviewer is correct that inoculation protocol modifies the interactions, and this is precisely the point: sequential inoculation deliberately introduces a temporal offset that grants the first-inoculated species a numerical and metabolic head start; while legitimate and industrially relevant, this is a fundamentally different question, whose dynamics reflect priority effects and imposed timing rather than the intrinsic co-occurring interactions we set out to isolate. Adopting it would confound the very effect the study is designed to resolve.
about the methodologies to track the yeasts dynamics, ARISA is amplifying rRNA genes which are known to be stable also when the cell is dying. Thereby the results of the dynamics reported in the study may be biased by the amplification of dead cells, which are detected even if they are not alive. For this reason, I strongly suggest integrating the data obtained by WLN counts with those of ARISA because the possibility to differentiate the species based on their colony topomorphology can support better the observations.	We thank the reviewer for this comment. This is a fair methodological point, and the requested cross-validation is already part of the study. We acknowledge in the manuscript — as is well recognized for DNA-based ARISA — that the method cannot by itself distinguish metabolically active from non-viable cells; for precisely this reason we independently tracked the consortia by viable plate counts on WL nutrient (WLN) medium, differentiating species by colony morphology (line 234: "through determination of the viable culturable population on differential media (Figure S3). A common succession pattern was observed in all consortia… "). We would emphasize that our interpretation rests on the relative succession patterns across consortia of differing composition, rather than on absolute population values, and it is precisely this comparative framing that makes the approach robust to the limitation the reviewer raises. The plating data (Figure S3) show population trajectories concordant with the ARISA-based dynamics: the two independent methods agree on the direction and pattern of the successional shifts, which justifies interpreting the ARISA signal as genuine community succession pattern rather than persistent dead-cell DNA. Nonetheless, we have added a clarification of this limitation to the Population dynamics section of the Materials and Methods
from the point of view of presenting the methodology used, it would better to clearly present in the main body of the paper because having relevant information in the supplementary material does not help.	We may have misunderstood this comment, as the methodological information necessary for the study is presented in the main body, with only supporting detail in the Supplementary Material. We have nonetheless reviewed the placement of methodological content and confirmed that all information essential to interpreting the results appears in the main text. We would welcome specific guidance from the reviewer if a particular element was felt missing.

Reviewer 1	Response
I would asl argue the selection of the term "drop-out". While the strain substitution is clear because it describes well that in the different consortia some strains have been left out, the drop-out species is a bit confusing because it gives a dynamic expectation to the reader, In other words, with the term drop-out, I was expecting some species to drop-out during fermentation, while what has been planned is consortia which contained or not certain species right from the beginning, thereby they were not dropping out. Please revise the term.	We appreciate the reviewer's concern. The term "drop-out" is used here in the established sense of a community-assembly design in which defined members are omitted from the assembled consortium, rather than implying loss during fermentation. Indeed, the term is defined explicitly at first use (line 111), where drop-out consortium are described as those in which each species was removed from the consortium in turn.
Also, I do not like the term "natural" throughout the manuscript because it implies that there are wine fermentations that are not natural. Probably spontaneous is a better term.	Thank you and agreed. We have replaced "natural" with "spontaneous" where it refers to uninoculated or wild-flora fermentations, which is the more precise and conventional term, and have verified that the remaining uses are unambiguous.
No discussion about acetic acid in the metabolites section. Please consider it as it was done for lactic acid (direct link to *H. uvarum*).	Thank you for your advice. We appreciate the suggestion and have expanded the metabolites discussion, while making an important distinction explicit. Lactic acid is an unusually clean tracer in this system: within our consortium it derives essentially from a single producer, *L. thermotolerans*, which is why it can be linked directly to one species and discussed independently. Acetic acid is not equivalent — it is produced by multiple community members (*H. uvarum* among them, but also as a general product of yeast oxidative and stress metabolism, including by *S. cerevisiae*), so its consortium-level concentration reflects a composite of overlapping contributions and cannot be attributed to *H. uvarum* alone. That said, we agree this point warranted fuller treatment, and together with the second comment above, we have expanded the metabolites discussion at the community level.
L491, while in agitation? Wine fermentation are carried our mostly static (apart from the pump overs).	We confirm this reflects a deliberate design choice rather than an oversight: agitation was applied to ensure homogeneity across the fermentation volume, which was necessary for representative and reproducible sampling in this experimental system, and applies uniformly across all treatments, so it does not confound the species- and strain-level comparisons that are the focus of this study.
Two important pints that have been missing in the introduction and discussion as factors influencing species and strains dynamics during fermentation where an established and emerging body of knowledge is present: cell-to-cell interaction and aminoacid preference of consumption between non Sacch and Sacch. Those can be factors affecting the outcomes described in the study, as well.	We thank the reviewer for these suggestions. We fully agree that contact-dependent cell-to-cell interactions and different preferences in amino acid consumption between non-Saccharomyces and Saccharomyces species are significant factors shaping wine yeast interactions. Relevant literature was indeed cited in the 3rd paragraph of introduction to cover these aspects. The present study, however, was designed to resolve species- and strain-specific consortium outcomes at the phenotypic level, and does not include the targeted measurements that would allow these mechanisms to be

Reviewer 1	Response
	assessed directly in community level; we have therefore been careful not to extend our interpretation beyond what the data support. We nonetheless agree that relevant knowledge should be discussed in the text, and have added relevant information in the discussion section, together with an explicit acknowledgment in the limitations paragraph.

Reviewer 2	Response
(Overall) Natural fermentation processes are driven by mixed and continuously changing communities (consortia) of microorganism. The alcoholic fermentation of grape juice, in which yeast are the major players, is no exception. The Stellenbosch team has been studying the population dynamics of wine-fermenting yeast communities since years. This manuscript reports on their project aimed to investigate the interactions of wine-born strains of five species in synthetic communities during the fermentation of a synthetic grape must. They investigated the consequences of dropping out of species from the consortia and replacing strains with conspecific strains. Their results show that in the experimental setting used in the study, each species had a significant effect on the structure and performance of the community, and its strains only fine-tuned the effect. This well-organised, well-written and very detailed study confirms the importance of ecological interactions between species. My only comment follows.	Thank you very much for your positive assessment of the study.
From the description of the experiments and the interpretation of the results, it seems that the dynamics of the populations was determined by the competition of the yeast strains. Four of the five species selected for this study are known to have antagonistic strains that adversely affect to growth/viability of other yeasts by releasing specific metabolites and/or secreting proteinaceous agents (e.g. killer factors). The authors could perhaps address the possible involvement of antagonism in the "ecological interactions" or why it can be ruled out in the consortia under investigation.	Thank you very much for your valuable suggestion, which we agree strengthens the framing of the interactions. We agree that direct antagonism — including killer-toxin activity and other secreted inhibitory agents — is a plausible contributor to the interactions observed, and we have added a short discussion acknowledging it as one candidate mechanism underlying the species- and strain-dependent effects. At the same time, we are careful not to overclaim: the study was not designed to measure killer phenotypes or toxin activity directly, so we can neither attribute specific outcomes to antagonism nor formally rule it out at this stage. We therefore note this openly and identify targeted assays of killer activity and secreted inhibitors as a great interest in the mechanistic follow-up study.

We greatly thank the editor for the positive assessment of our revised manuscript. Following the suggestion, we have expanded the title to identify the experimental system more specifically. The revised title reads "**Species and strain-specific phenotypes shape performance and output of a five-species yeast consortium**". The running title has also been changed accordingly to "**Species and strain effects in yeast consortia**".

No other changes have been made to the manuscript.

Re: Spectrum02819-26R1 (Species and strain-specific phenotypes shape performance and output of a five-species yeast consortium)

Dear Prof. Florian F Bauer:

Your manuscript has been accepted, and I am forwarding it to the ASM production staff for publication. Your paper will first be checked to make sure all elements meet the technical requirements. ASM staff will contact you if anything needs to be revised before copyediting and production can begin. Otherwise, you will be notified when your proofs are ready to be viewed.

Data Availability: ASM policy requires that data be available to the public upon online posting of the article, so please verify all links to sequence records, if present, and make sure that each number retrieves the full record of the data. If a new accession number is not linked or a link is broken, provide production staff with the correct URL for the record. If the accession numbers for new data are not publicly accessible before the expected online posting of the article, publication may be delayed; please contact ASM production staff immediately with the expected release date.

Publication Fees: For information on publication fees and which article types have charges, please visit our website. We have partnered with Copyright Clearance Center (CCC) to collect author charges. If fees apply to your paper, you will receive a message from no-reply@copyright.com with further instructions. For questions related to paying charges through RightsLink, please contact CCC at ASM_Support@copyright.com or toll free at +1-877-622-5543. CCC makes every attempt to respond to all emails within 24 hours.

ASM Membership: Corresponding authors may join or renew ASM membership to obtain discounts on publication fees. Need to upgrade your membership level? Please contact Customer Service at Service@asmusa.org.

Embargo Policy: A press release may be issued as soon as the manuscript is posted on the Spectrum Latest Articles webpage. The corresponding author will receive an email with the subject line "ASM Journals Author Services Notification" when the article is available online.

Cover Image Submissions: If you would like to submit a potential Cover Image, please email a file and a short legend to spectrum@asmusa.org. Please note that we can only consider images that (i) the authors created or own and (ii) have not been previously published. By submitting, you agree that the image can be used under the same terms as the published article. Image File requirements: TIF/EPS, 7.5 inches wide by 8.25 inches tall (at least 2,250 pixels wide by 2,475 pixels tall), minimum 300 dpi resolution (600 dpi preferred), RGB, and no figure elements, e.g., arrows or panel labels. The legend should be a short description of the image, 1-2 sentences recommended. Please use the ASM Journals Cover Image Sizing Tool (https://journals.asm.org/pb-assets/html/asm-cover-sizing-tool.html) to ensure that your proposed cover image meets our size requirements.

The ASM Journals program strives for constant improvement in our submission and publication process. Please tell us how we can improve your experience by taking this quick Author Survey.

Thank you for submitting your paper to Spectrum.

Sincerely,
Blaire Steven
Editor
Microbiology Spectrum
