# Supplementary material for: Species and strain-specific phenotypes shape performance and output of a five-species yeast consortium
Source: Microbiol Spectr. 2026 Sep 24;14(11):e02819-26. doi: 10.1128/spectrum.02819-26 (PMC13729150; doi:10.1128/spectrum.02819-26)
Supplement: Supplementary file 2 [file spectrum.02819-26-s0001.pdf]

Figures:

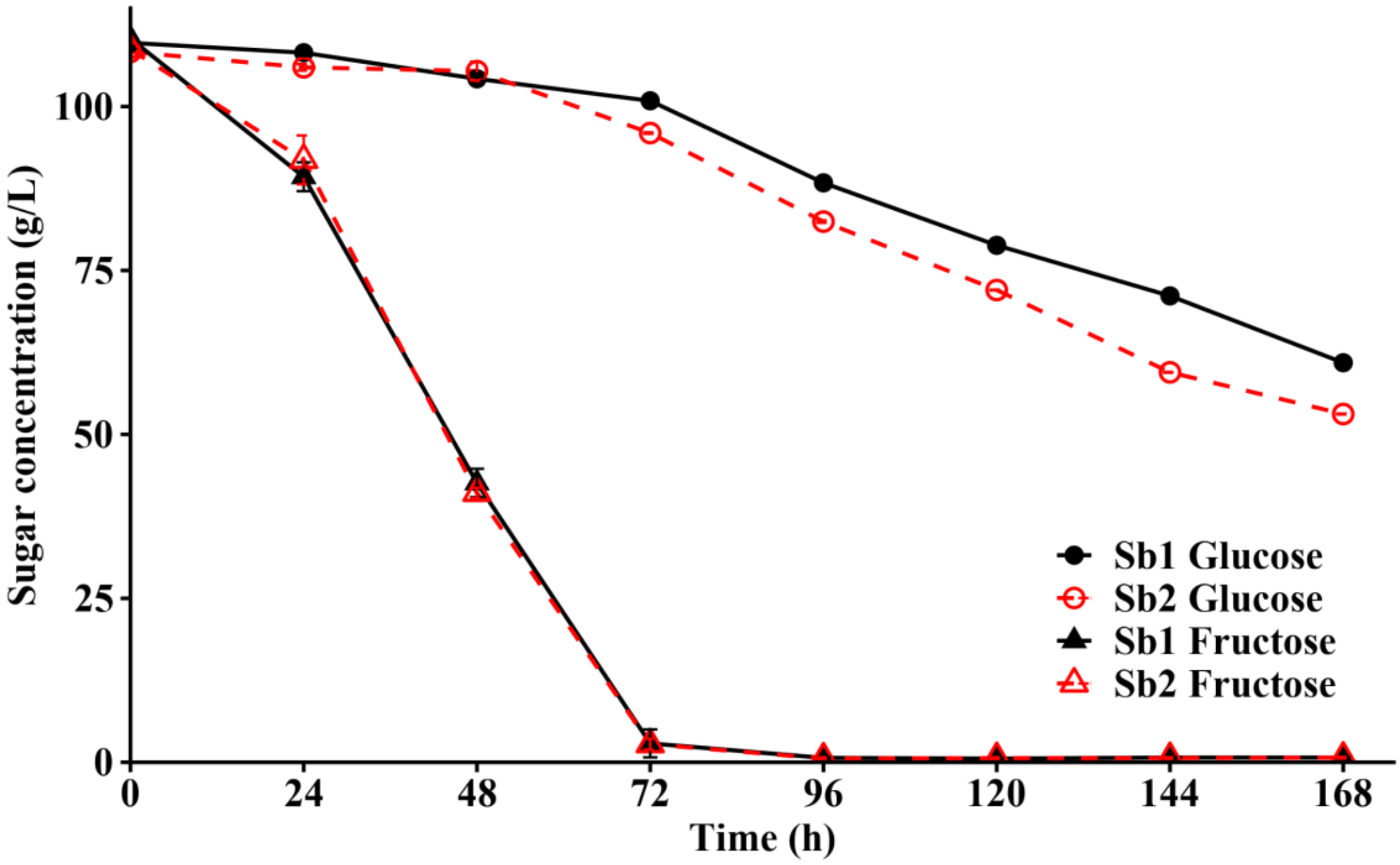

Figure S 1. Glucose and fructose consumption by two *S. bacillaris* strains cultivated in synthetic grape must (SGM). Sb1 glucose (black filled circle, solid line), Sb2 glucose (red open circle, dashed line), Sb1 fructose (black filled triangle, solid line), Sb2 fructose (red open triangle, dashed line). Error bars represent standard deviation (SD) of three independent replicates.

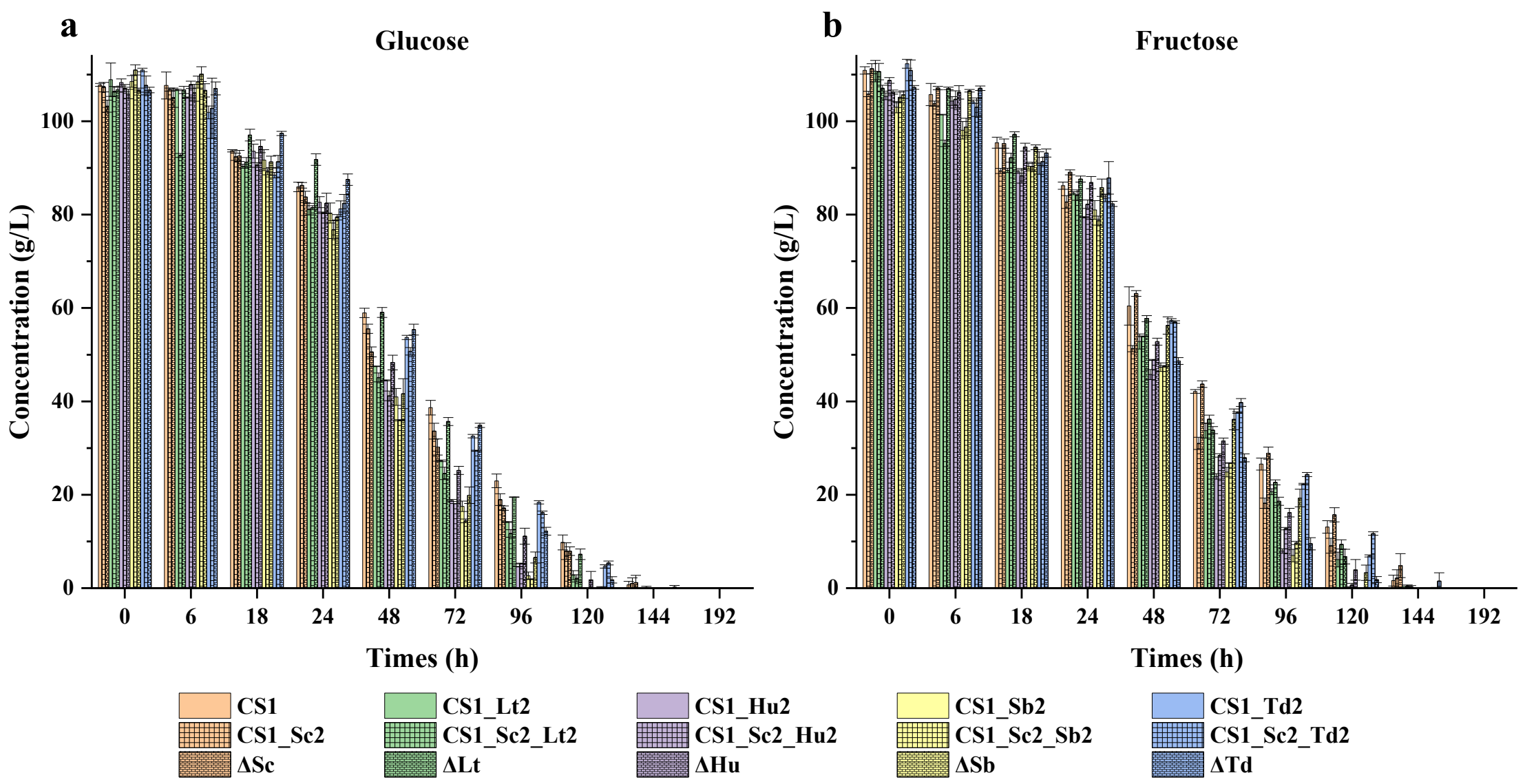

Figure S 2. Residual glucose and fructose concentrations across fifteen synthetic yeast consortia. Glucose and fructose concentrations were determined enzymatically at each sampling point throughout the 192 h fermentation. (a) Residual glucose. (b) Residual fructose. Bar color encodes the perturbed species (CS1, orange; Lt2, green; Hu2, purple; Sb2, yellow; Td2, blue). Bar fill encodes perturbation status (solid, Sc1-background consortia; crosshatch, Sc2-background consortia; dense crosshatch, single-species drop-out consortia). Bars represent the mean of three biological replicates; error bars indicate ± SD.

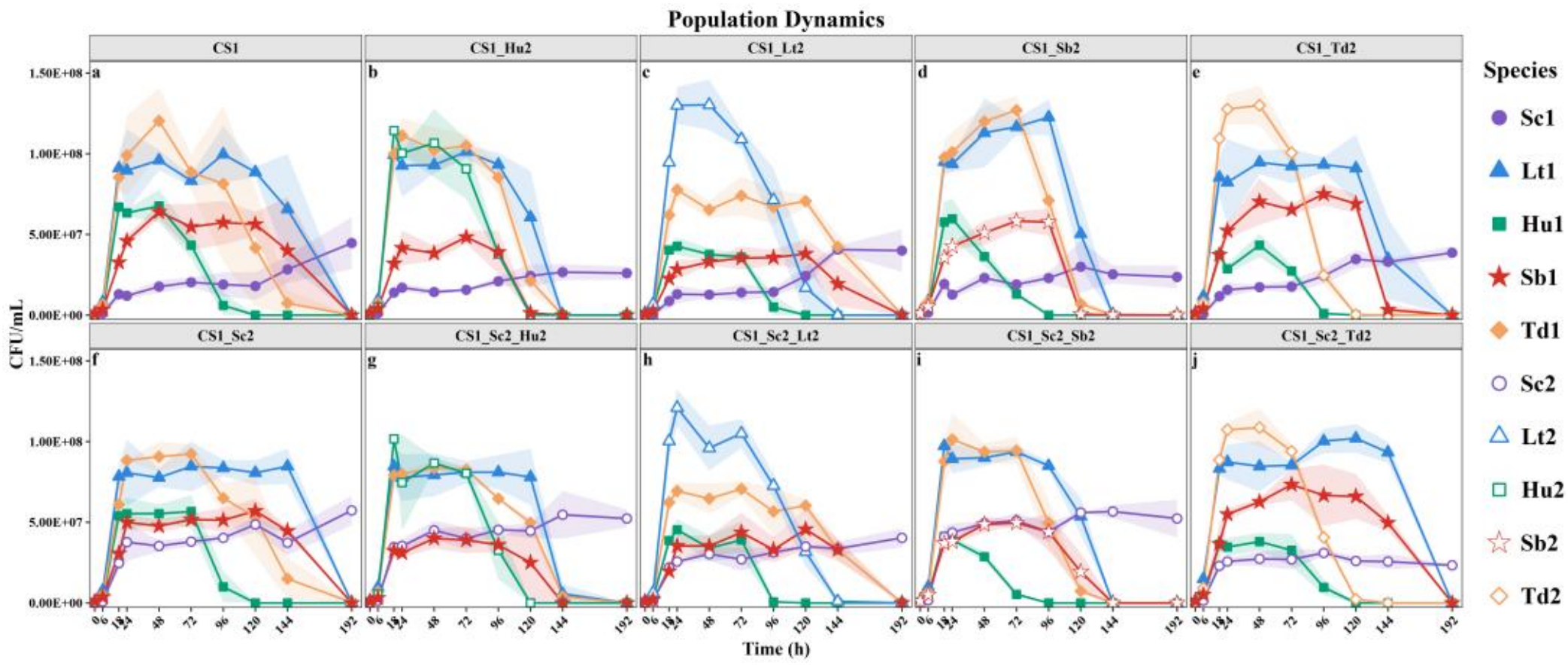

Figure S 3. Viable culturable cell counts showing the growth dynamics of ten strain-switch consortia determined by plating. (a–e) Consortia on the Sc1 background. (f–j) Consortia on the Sc2 background. Each panel represents one consortium. Species are distinguished by color and marker (Sc, purple circles; Lt, blue triangles; Hu, green squares; Sb, red stars; Td, orange diamonds); filled markers denote strain 1 and open markers denote strain 2. Curves represent the mean of three biological replicates; shaded bands indicate ± SD.

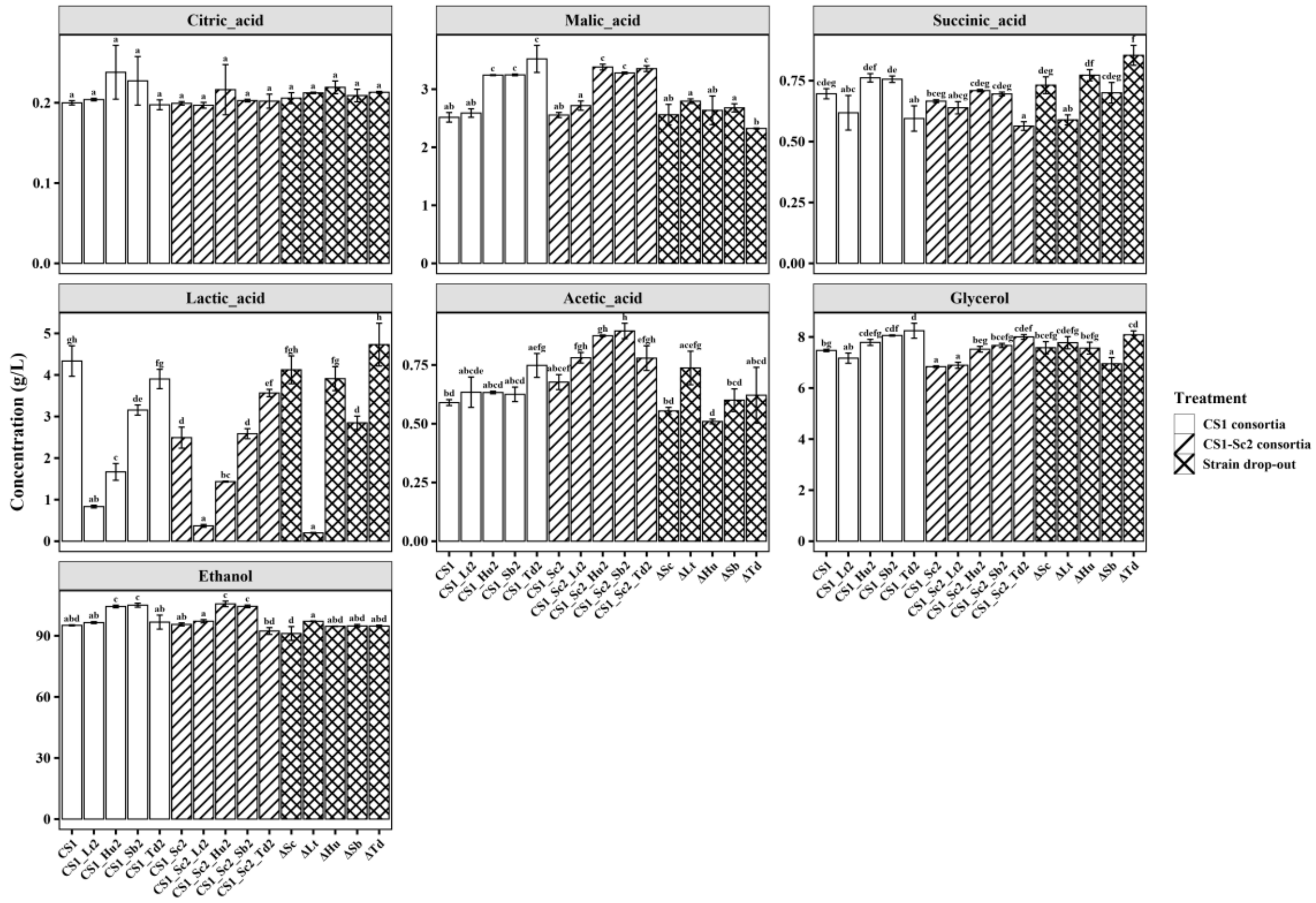

Figure S 4. End-of-fermentation concentrations of primary metabolites across fifteen synthetic yeast consortia. Concentrations of malic acid, lactic acid, citric acid, succinic acid, acetic acid, glycerol, and ethanol were determined by HPLC at the end of the 192 h fermentation. Bars represent the mean of three biological replicates, with individual replicate values overlaid; error bars indicate ± SD. Bar fill encodes perturbation status (open, Sc1-background consortia; diagonal stripes, Sc2-background consortia; crosshatched, single-species drop-out consortia). Lowercase letters indicate Tukey HSD groupings (α = 0.05) within each metabolite panel.

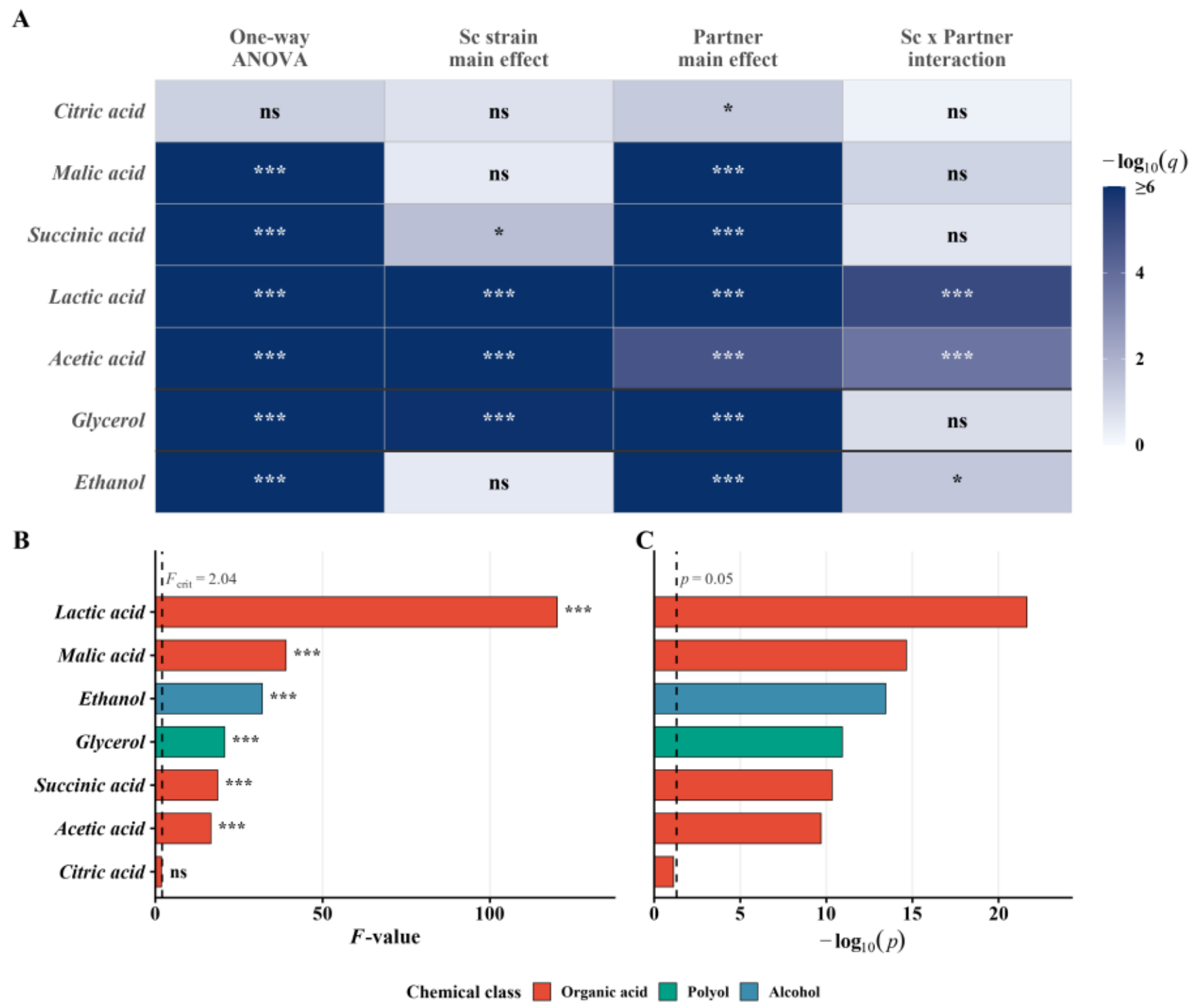

Figure S 5. ANOVA-based statistical overview of HPLC-quantified metabolites across the synthetic wine consortia. (A) Heatmap summarizing the significance of consortium-driven variation for seven HPLC-quantified metabolites (rows, grouped by chemical class; horizontal lines separate classes) under four tests (columns). Glucose and fructose were excluded from the analysis owing to their negligible residual concentrations in the finished (dry) wines. Column 1 (One-way ANOVA) tests whether each metabolite differs across all 15 consortia (single factor = consortium; df = 14, 30). Columns 2–4 are derived from a single two-way ANOVA fitted only to the substitution subset, i.e. the ten CS1-based consortia forming a balanced 2 × 5 design (Sc strain, Sc1 vs Sc2; partner species, none/Lt2/Hu2/Sb2/Td2; n = 3 per cell). Column 2 (Sc strain main effect) tests the average effect of replacing the *Saccharomyces cerevisiae* strain (Sc1 → Sc2); column 3 (Partner main effect) tests the average effect of partner-species identity; column 4 (Sc × Partner interaction) tests whether the effect of the Sc strain depends on partner identity. The five drop-out (Δ) consortia contribute to column 1 only and were not included in the two-way model. Cell shading encodes $-\log_{10}(q)$, where q is the Benjamini–Hochberg false-discovery-rate-adjusted p-value computed separately within each column across the seven metabolites; shading is capped at $-\log_{10}(q) = 6$. Symbols indicate significance after FDR correction: *** $q < 0.001$, ** $q < 0.01$, * $q < 0.05$, ns = not significant. (B, C) One-way ANOVA results for the same seven metabolites, ordered by F-value and colored by chemical class. (B) F-values; the dashed line marks the critical F at $\alpha = 0.05$ ($F_{(crit)} = 2.04$; df = 14, 30). (C) $-\log_{10}(p)$; the dashed line marks $p = 0.05$. Significance symbols in (B) reflect FDR-adjusted q-values, consistent with panel A.

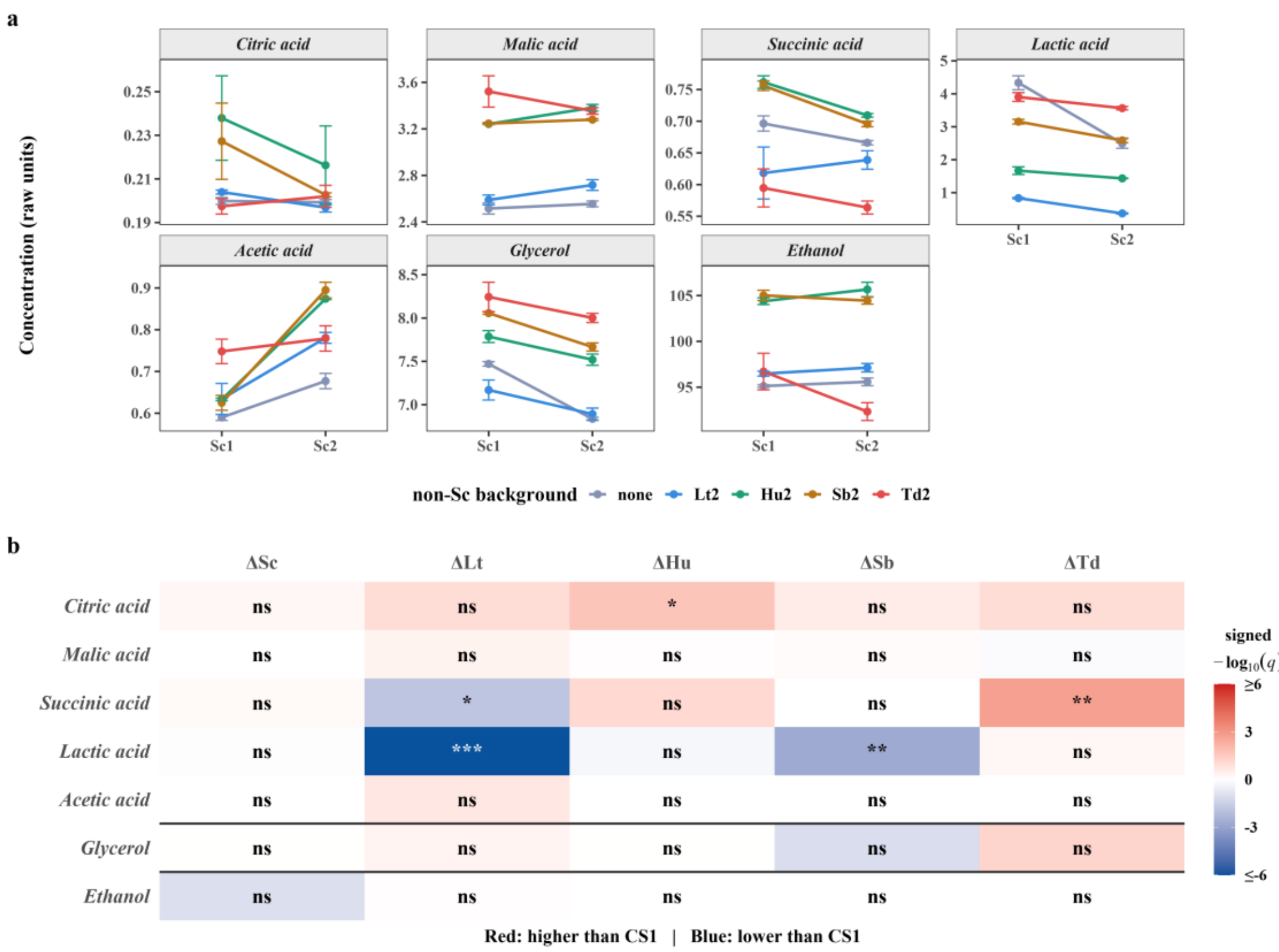

Figure S 6. Strain-by-partner interactions and the effects of single-species drop-out on the primary metabolite profile. (a) Interaction plots for each of the seven HPLC-quantified metabolites, derived from the two-way ANOVA fitted to the ten substitution consortia (the balanced 2 × 5 design); glucose and fructose were excluded owing to their negligible residual concentrations in the dried wines. For each non-*Saccharomyces* background, the mean concentration (raw units; symbols, mean ± standard error of three biological replicates) is shown for the Sc1 and Sc2 backgrounds and joined by a line, colored by the non-*Saccharomyces* background. Parallel lines indicate that the effect of switching the *S. cerevisiae* strain is consistent across backgrounds (no interaction), whereas non-parallel lines indicate that the strain effect depends on the co-present non-*Saccharomyces* species (a significant interaction). This panel visualizes the interaction term of the two-way ANOVA reported in Figure S5 (column 4). (b) Heatmap of Dunnett's many-to-one comparisons, in which each single-species drop-out consortium (ΔSc, ΔLt, ΔHu, ΔSb, ΔTd) is compared with the control community (CS1). Rows are seven metabolites, grouped by chemical class (black lines separate classes); columns are the drop-out consortia. Cell color encodes the signed $-\log_{10}(q)$, where red denotes a higher concentration and blue denotes a lower concentration relative to CS1, and the magnitude reflects the Benjamini–Hochberg-adjusted significance. Symbols denote significance after FDR correction (*** $q < 0.001$, ** $q < 0.01$, * $q < 0.05$; ns, not significant). Whereas the two-way ANOVA in (a) resolves the effect of substituting one strain for its counterpart, the drop-out comparisons in (b) test the consequence of removing a species entirely from the community.

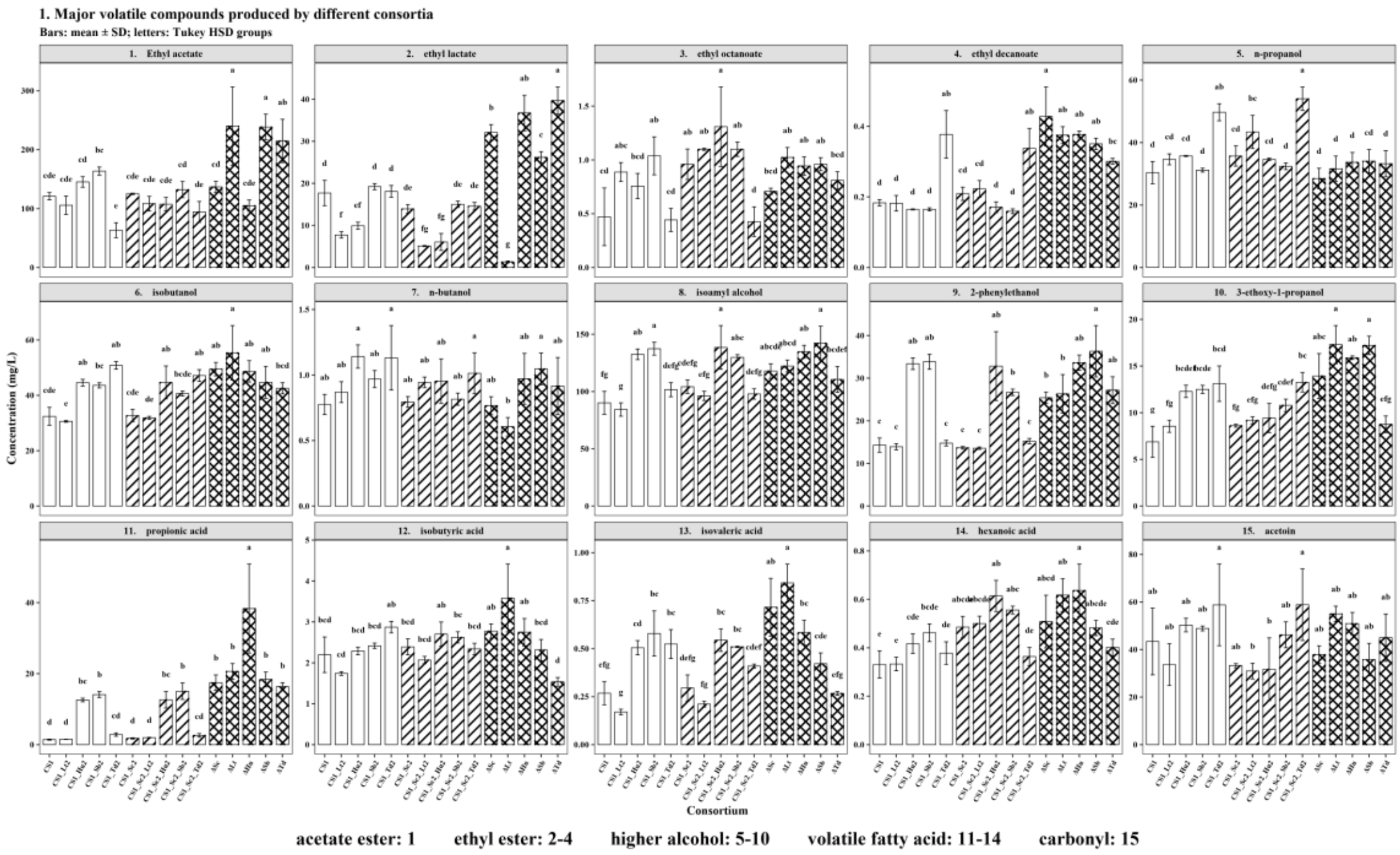

Figure S 7. End-of-fermentation concentrations of fifteen major volatile compounds across fifteen synthetic yeast consortia. Concentrations (mg L^{-1}) were determined by GC-FID at the end of the 192 h fermentation. Bars represent the mean of three biological replicates, with individual replicate values overlaid; error bars indicate ± SD. Bar fill encodes perturbation status (open, Sc1-background consortia; diagonal stripes, Sc2-background consortia; crosshatched, single-species drop-out consortia). Lowercase letters indicate Tukey HSD groupings (α = 0.05) within each compound panel.

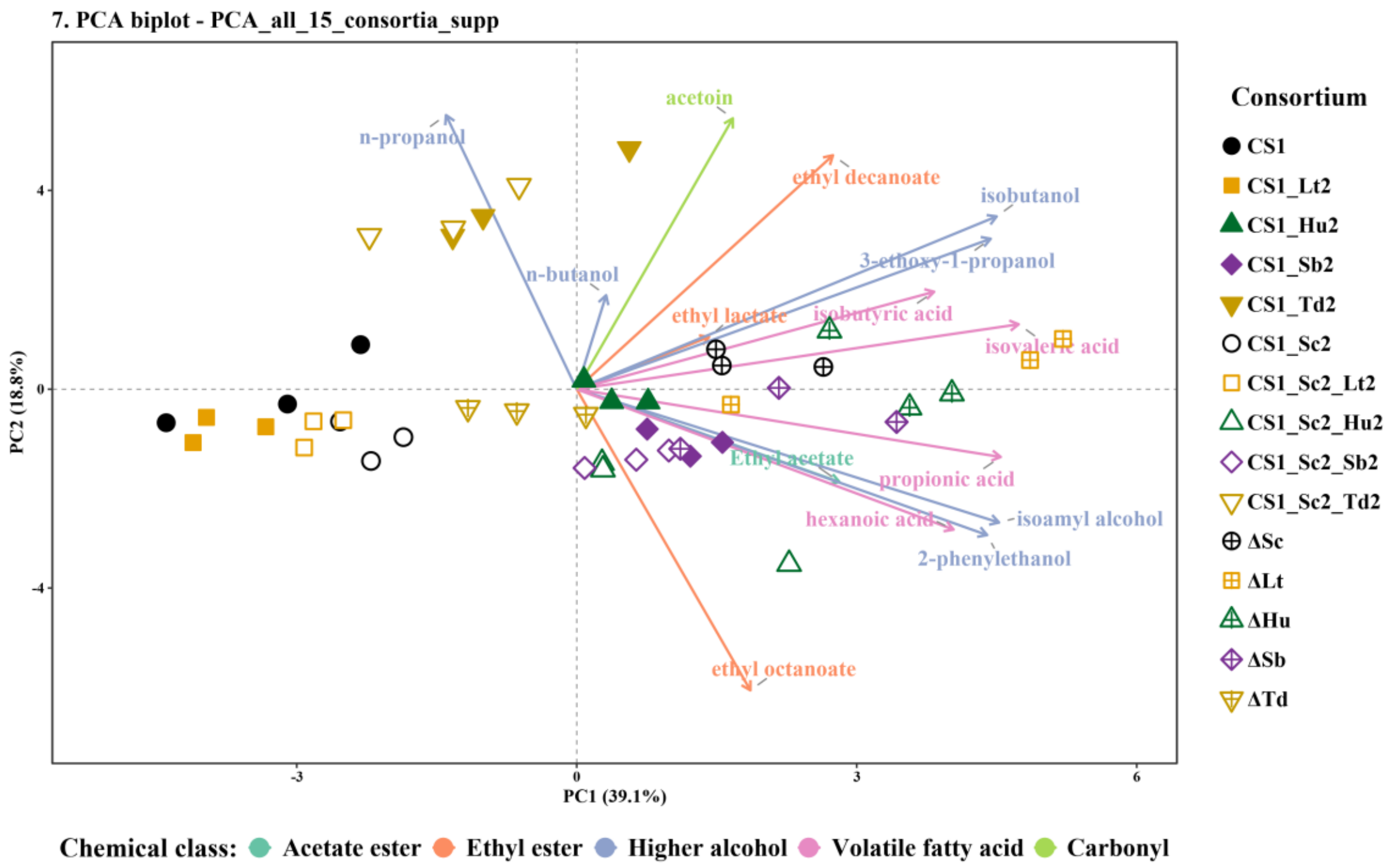

Figure S 8. Global principal component analysis of major volatile profiles across all fifteen consortia. PCA was performed on end-of-fermentation concentrations of fifteen major volatile compounds determined by GC-FID, incorporating all fifteen consortia simultaneously. Each point represents one biological replicate, with three replicates shown per consortium; volatile loading vectors are overlaid. The percentage of total variance explained is indicated on each axis.

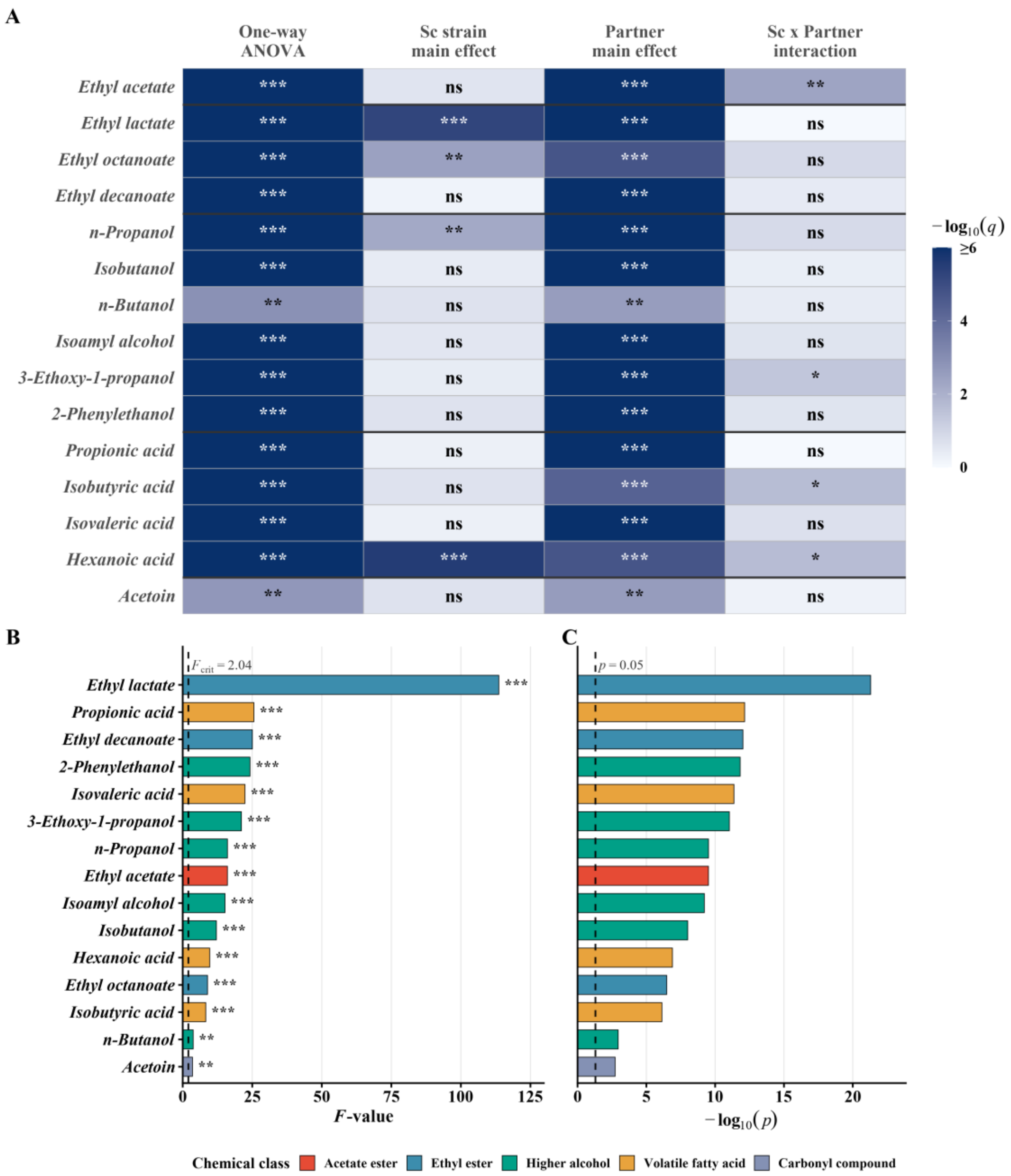

Figure S 9. ANOVA-based statistical overview of major volatile compounds across the synthetic wine consortia. (A) Heatmap summarizing the significance of consortium-driven variation for each of the 15 major volatile compounds (rows, grouped by chemical class; horizontal lines separate classes) under four tests (columns). Column 1 (One-way ANOVA) tests whether each compound differs across all 15 consortia (single factor = consortium; df = 14, 30). Columns 2–4 are derived from a single two-way ANOVA fitted only to the substitution subset, i.e. the ten CS1-based consortia forming a balanced 2 × 5 design (Sc strain, Sc1 vs Sc2; partner species, none/Lt2/Hu2/Sb2/Td2; n = 3 per cell). Column 2 (Sc strain main effect) tests the average effect of replacing the *Saccharomyces cerevisiae* strain (Sc1 → Sc2); column 3 (Partner main effect) tests the average effect of partner-species identity; column 4 (Sc × Partner interaction) tests whether the effect of the Sc strain depends on partner identity. The five drop-out (Δ) consortia contribute to column 1 only and were not included in the two-way model. Cell shading encodes

$-\log_{10}(q)$, where q is the Benjamini–Hochberg false-discovery-rate-adjusted p-value computed separately within each column across all compounds; shading is capped at $-\log_{10}(q) = 6$. Symbols indicate significance after FDR correction: *** $q < 0.001$, ** $q < 0.01$, * $q < 0.05$, ns = not significant. (B, C) One-way ANOVA results for the same compounds, ordered by F-value and colored by chemical class. (B) F-values; the dashed line marks the critical F at $\alpha = 0.05$ ($F_{(crit)} = 2.04$; df = 14, 30). (C) $-\log_{10}(p)$; the dashed line marks $p = 0.05$. Significance symbols in (B) reflect FDR-adjusted q-values, consistent with panel A.

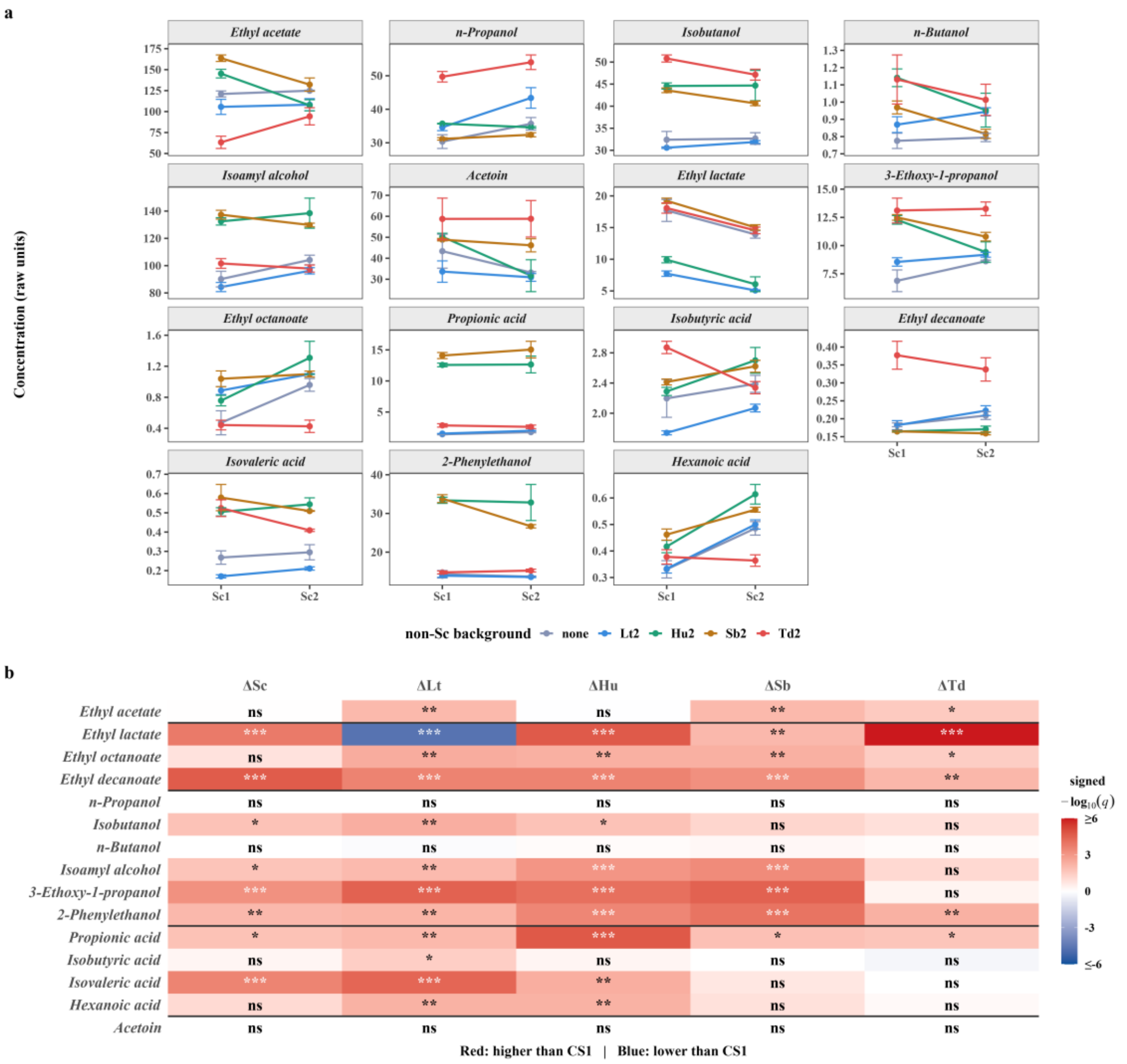

Figure S 10. Strain-by-partner interactions and the effects of single-species drop-out on the major volatile profile. (a) Interaction plots for each of the fifteen major volatile compounds, derived from the two-way ANOVA fitted to the ten substitution consortia (the balanced 2 × 5 design). For each non-*Saccharomyces* background, the mean concentration (raw units; symbols, mean ± standard error of three biological replicates) is shown for the Sc1 and Sc2 backgrounds and joined by a line; lines are colored by the non-*Saccharomyces* background. Parallel lines indicate that the effect of switching the *S. cerevisiae* strain is consistent across backgrounds (no interaction), whereas non-parallel lines indicate that the strain effect depends on the co-present non-*Saccharomyces* species (a significant interaction). This panel visualizes the interaction term of the two-way ANOVA reported in Figure S9 (column 4). (b) Heatmap of Dunnett's many-to-one comparisons, in which each single-species drop-out consortium (ΔSc, ΔLt, ΔHu, ΔSb, ΔTd) is compared with the complete community (CS1). Rows are the fifteen compounds, grouped by chemical class (black lines separate classes); columns are the drop-out consortia. Cell color encodes the signed $-\log_{10}(q)$, where red denotes a higher concentration and blue a lower concentration relative to CS1, and the magnitude reflects the Benjamini–Hochberg-adjusted significance. Symbols denote significance after FDR correction (*** $q < 0.001$, ** $q < 0.01$, * $q < 0.05$; ns, not significant).

Whereas the two-way ANOVA in (a) resolves the effect of substituting one strain for its counterpart, the drop-out comparisons in (b) test the consequence of removing a species entirely from the community.

Tables:

Table S 1 Inoculation strategy for each strain in consortium

Strains	Inoculation (cells/mL)
Sc1	2×10^5
Sc2	2×10^5
Lt1	1×10^6
Lt2	1×10^6
Hu1	5×10^5
Hu2	5×10^5
Sb1	1×10^6
Sb2	1×10^6
Td1	1×10^6
Td2	1×10^6

Table S 2. Calibration curves between OD and cells/mL

Strains	Calibration Curve
Sc1 Pinnacle	$y = 2.33\times10^6x + 6115.31$
Sc2 Y1464	$y = 3.88\times10^6x - 51033.80$
Lt1 Laktia	$y = 5.26\times10^6x - 22736.02$
Lt2 Concerto	$y = 3.85\times10^6x - 45785.45$
Hu1 Y861	$y = 1.32\times10^7x - 101551.00$
Hu2 Y966	$y = 1.24\times10^7x - 53625.90$
Sb1 Y943	$y = 1.00\times10^7x - 84201.00$
Sb2 Y547	$y = 1.21\times10^7x - 5764.82$
Td1 NJ783	$y = 6.61\times10^6x + 44731.60$
Td2 biodiva	$y = 5.82\times10^6x + 99670.10$
